# Controlled Release of Microorganisms from Engineered Living Materials

**DOI:** 10.1021/acsami.5c11155

**Published:** 2025-07-08

**Authors:** Manivannan Sivaperuman Kalairaj, Iris George, Sasha M. George, Sofía E. Farfán, Yoo Jin Lee, Laura K. Rivera-Tarazona, Suitu Wang, Mustafa K. Abdelrahman, Seelay Tasmim, Asaf Dana, Philippe E. Zimmern, Sargurunathan Subashchandrabose, Taylor H. Ware

**Affiliations:** † Department of Biomedical Engineering, 14736 Texas A&M University, College Station, Texas 77843, United States; ‡ Department of Veterinary Pathobiology, College of Veterinary Medicine and Biomedical Sciences, Texas A&M University, College Station, Texas 77843, United States; § Department of Materials Science and Engineering, Texas A&M University, College Station, Texas 77843, United States; ∥ School of Engineering, 28033 Pontificia Universidad Católica de Chile, Santiago 7820436, Chile; ⊥ Department of Urology, The University of Texas Southwestern Medical Center, Dallas, Texas 75390, United States

**Keywords:** controlled release, engineered living materials, probiotics, sustained release, zero-order release

## Abstract

Probiotics offer therapeutic benefits by modulating the local microbiome, the host immune response, and the proliferation of pathogens. Probiotics have the potential to treat complex diseases, but their persistence or colonization is required at the target site for effective treatment. Although probiotic persistence can be achieved by repeated delivery, no biomaterial that releases clinically relevant doses of metabolically active probiotics in a sustained manner has been previously described. Here, we encapsulate stiff probiotic microorganisms within relatively less stiff hydrogels and show a generic mechanism where these microorganisms proliferate and induce hydrogel fracture, resulting in microbial release. Importantly, this fracture-based mechanism leads to microorganism release with zero-order release kinetics. Using this mechanism, small (∼1 μL) engineered living materials (ELMs) release >10^8^ colony-forming-units (CFUs) of *Escherichia coli* in 2 h. This release is sustained for at least 100 days. Cell release can be varied by more than 3 orders of magnitude by varying initial cell loading and modulating the mechanical properties of the encapsulating matrix. As the governing mechanism of microbial release is entirely mechanical, we demonstrate the controlled release of model Gram-negative, Gram-positive, and fungal probiotics from multiple hydrogel matrices.

## Graphical abstract

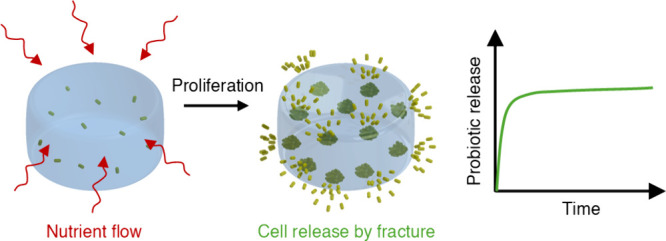

## 1 Introduction

Controlled release systems of therapeutic agents aim to deliver a predefined dose at a target site over a sustained period of time.^1–5^ Such systems decrease side effects and the number of administrations required for treatment compared to systemic delivery.^2^ It is often desirable, but difficult, to generate time-invariant (zero-order) release profiles with the delivered dosage in the therapeutic range.^1,2,6,7^ Although fundamental relationships that govern the release kinetics of small molecules and macromolecules from synthetic materials have been developed,^1,2^ the governing mechanisms that control the release of microorganisms in a controlled and sustained manner have not been established.

Probiotics are live microorganisms^8,9^ that, when dosed appropriately, offer many health benefits such as modulating the local microbiome,^9–13^ the host immune response,^14,15^ and the proliferation of pathogens.^9,16^ Probiotics have been proposed to provide therapeutic benefits for constipation,^17^ diabetes mellitus,^18^ *Helicobacter pylori* infections,^11^ inflammatory bowel disease,^19^ irritable bowel syndrome,^19^ *Clostridium difficile* infections,^15^ urinary tract infections,^10^ and vaginal infections.^14^ Although probiotics aid in the treatment of complex diseases, several clinical studies show conflicting results,^20,21^ which could be due to the probiotic dosage, mode/timings of administration, strain used, or inclusion of prebiotics.^22^ A key challenge to the clinical utility of probiotics is the need to administer probiotics in a metabolically active state at clinically relevant dosage.^8^

Various encapsulation technologies have been developed to increase the survival of probiotics and shield them from harsh environments.^3,5,23,24^ For example, in orally delivered probiotics, encapsulation protects the ingested cells from the harsh environment of the stomach.^25,26^ However, even if cells are successfully delivered, the persistence of probiotics, an important requirement to achieve beneficial effects,^25,27^ is often not observed in the GI tract or other niches.^28–31^ To circumvent this drawback, probiotic persistence can be achieved by promoting adhesion between the probiotic and mucus membranes^25^ by either engineering the microbe to enhance its adherence^31^ or coating it with mucoadhesins.^32^ For probiotics that lack adhesiveness, repeated administration of a prescribed dose of viable probiotics for a long period can promote colonization.^33^ While repeated administration is feasible for accessing some mucosal sites such as the gut and lower reproductive tract, it is a barrier for effective deployment of probiotic-based therapies in hard-to-access sites, such as the urinary bladder.^34^

Currently, controlled release of probiotics is achieved through the degradation of the encapsulating matrices^3–5,8^ or transport of probiotics through porous materials.^35^ Although these approaches mirror the mechanisms used for the controlled release of small molecules,^2^ they fail to release probiotics in a sustained manner.^3–5,8,35^ This is because unlike drug/therapeutic delivery systems where several days’ worth of drug/therapeutics can be loaded and released at the site in a sustained manner,^2^ probiotic transport through a solid material is limited due to the large size of cells in comparison to drugs/therapeutics,^2,36^ making it difficult to load and deliver many cells. Hence, to release high doses of probiotics in a sustained manner, we need a mechanism that allows local replication of probiotics at the target site, like an in situ probiotic factory.^37^

ELMs are composites frequently constructed by embedding living microorganisms into organic or inorganic matrices.^13,38–41^ ELMs are endowed with complex emergent functionality from the interplay between their living and nonliving components.^38,39,42^ The nonliving component of ELMs maintains the viability of microorganisms by facilitating the diffusion of water, nutrients, gases, and biomolecules.^38^ Notably, the properties of the nonliving matrix help modulate the interactions of the microbes with the surrounding environment.^40,43^ In many ELMs, microorganism escape is observed,^39,42,44,45^ and biocontainment platforms have been developed to prevent that escape.^46^ In the ELM field, microbial escape is typically considered a drawback as unwanted release of genetically modified microorganisms and unplanned growth in surroundings could be a source of future regulatory concerns,^47^ whereas, with common probiotics found in the population^43^ or used in food production,^48^ microbial release is not a concern. We have previously demonstrated that using ELMs, probiotics can be released from nonporous and nondegrading synthetic polymers.^43^ However, the mechanism of microbial escape is poorly understood and the controlled and sustained release of microorganisms has not been demonstrated. Precise control of microorganism escape could be exploited to realize a technological or clinical utility.

Herein, we elucidate a generic mechanism in which stiff microorganisms proliferating within relatively low elastic modulus hydrogels induce fracture in those hydrogels, causing microbial release. Surprisingly, this mechanism yields sustained release of microorganisms with zero-order release kinetics. The rate of microbial release is modulated by varying the initial microorganism loading, mechanical properties of the encapsulating hydrogels, and the shape and size of the ELMs. Furthermore, since this mechanism is entirely mechanically driven, this mechanism can be extended to several probiotics and synthetic polymers. Hence, we demonstrate the sustained release of a Gram-negative probiotic bacterium (*Escherichia coli* (*E. coli*) ABU 83972), a Gram-positive probiotic bacterium (*Lacticaseibacillus paracasei* (*L. paracasei*)), and a probiotic fungus (*Saccharomyces cerevisiae* (*S. cerevisiae*)) from two types of acrylic hydrogels.

## 2 Results and Discussion

### 2.1 Microorganisms Release from ELMs in a Sustained Manner

To make a probiotic factory and delivery mechanism, we synthesized ELMs with an encapsulated living probiotic, such as *E. coli* (ABU 83972), embedded within acrylic hydrogels. The hydrogels were prepared by free radical polymerization of 2-hydroxyethyl acrylate (HEA) (monomer) and *N*,*N*′-methylenebis­(acrylamide) (BIS) (cross-linker). When these ELMs are incubated in growth media, nutrients diffuse through the hydrogel matrices,^38^ allowing bacteria to proliferate within the hydrogel matrices, resulting in colony formation. It is known that colonies exert mechanical forces on the surrounding hydrogel during expansion.^40,49^ We hypothesized that upon reaching a certain volume, the colony induces a fracture in the hydrogels, causing bacterial release (Figure 1a,b). Incubating identical ELMs in nongrowth media such as saline does not allow cell growth; hence, no release is observed (Figure 1c). Moving ELMs that were first grown for 5 days into saline dramatically reduces cell release. These data collectively confirm the necessity of microbial proliferation to induce fractures and release microorganisms. Surprisingly, this mechanism leads to sustained release of *E. coli* with a zero-order release kinetics (Figure 1c). ELMs loaded with 1 × 10^4^ cells per μL of ELM release cells after 1 day of growth and release 4.03 ± 0.18 × 10^7^ colony-forming-units (CFUs) per μL of ELM in 2 h (Figure 1c and Supporting Information Figure S1). Moreover, once the ELMs reach steady-state release, the number of cells present in the ELMs does not change significantly during the 2 h of cell release (Figure 1d and Figure S2). As such, the cells present in the ELMs remain within the ELMs to facilitate sustained release.

**Figure 1:**
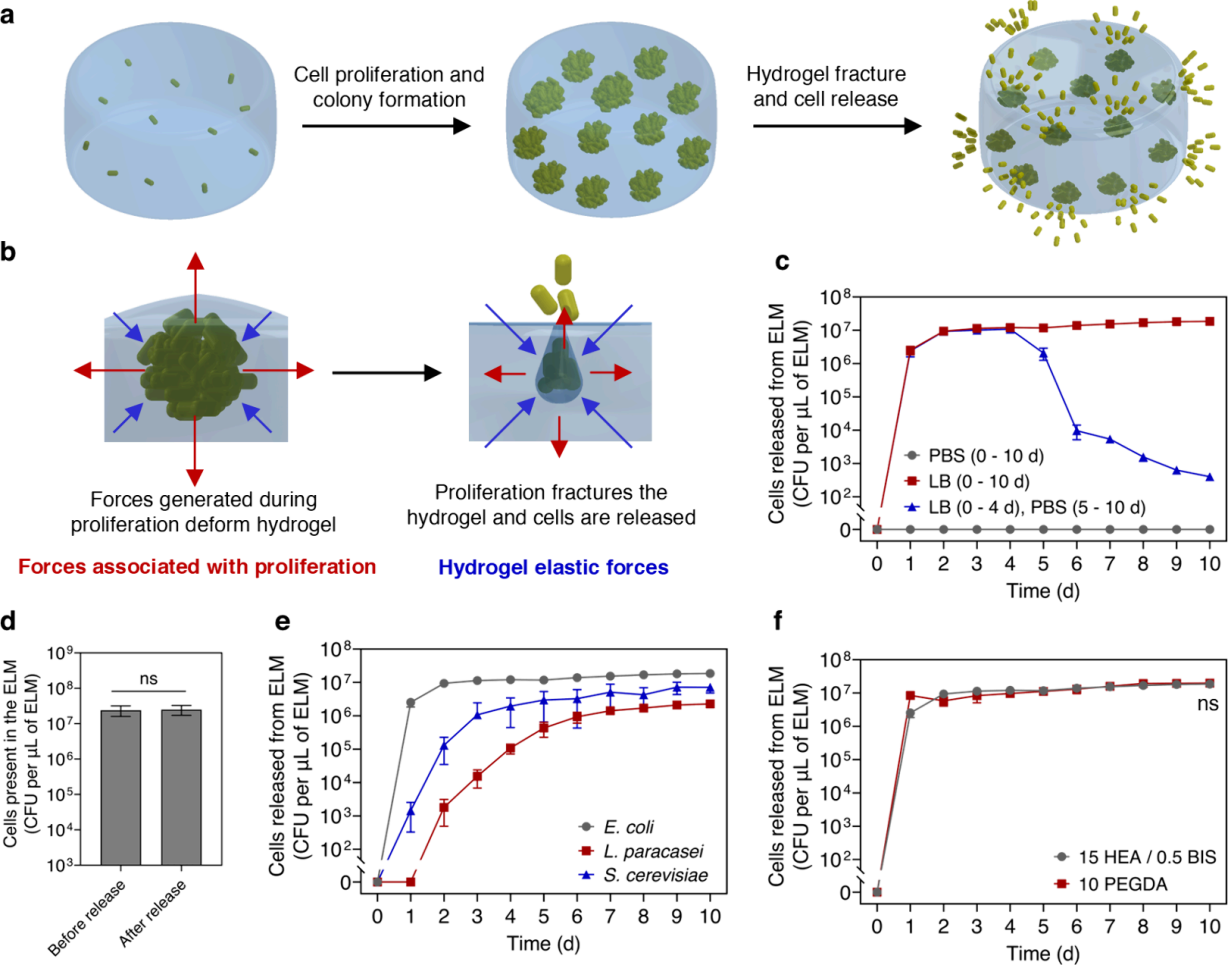
Sustained release of microorganisms from ELMs. (a) Schematic illustrating the sustained release of microorganisms from ELMs. Viable microorganisms encapsulated within the ELMs can proliferate and form colonies and can also be released. (b) Schematic illustrating the interplay between microbial proliferation forces and hydrogel elastic forces that induce fracture and cell release. (c) Cell release as a function of time when ELMs are incubated in LB media (nutrient media) and saline (PBS). (d) Number of cells present within the ELMs before and after 2 h of cell release (5 day grown ELMs). (e) Cell release as a function of time from ELMs loaded with different microorganisms (*E. coli* (gray), *L. paracasei* (red), and *S. cerevisiae* (blue)). (f) Cell release as a function of time from ELMs prepared with different types of hydrogels (HEA/BIS (gray) and PEGDA (red)). ELMs in panels c and d were prepared with a cell loading of 1 × 10^4^ *E. coli* ABU 83972 cells per μL of ELM and a hydrogel formulation of 15 HEA/0.5 BIS (wt %). All ELMs in panel e were prepared with a hydrogel formulation of 15 HEA/0.5 BIS. *E. coli*-loaded ELMs were prepared with 1 × 10^4^ *E. coli* cells per μL of ELM, *L. paracasei*-loaded ELMs were prepared with 1 × 10^3^ *L. paracasei* cells per μL of ELM, and *S. cerevisiae*-loaded ELMs were prepared with 1 × 10^3^ *S. cerevisiae* cells per μL of ELM. All ELMs in panel f were prepared with a cell loading of 1 × 10^4^ *E. coli* cells per μL of ELM. All data in panels c–f are presented as mean ± standard deviations (*n* = 3). Statistical analysis was performed by a two-tailed Student’s *t*-test. Not significant (ns) for *P* > 0.05.

The driving mechanism for cell release is mechanical; hence, this mechanism can be extended to several probiotics and synthetic polymers. Using the same hydrogel formulations, sustained release of *L. paracasei* and *S. cerevisiae* is observed (Figure 1e). In addition to HEA/BIS hydrogels, we also demonstrate a zero-order release of *E. coli* from ELMs with a different hydrogel matrix made from cross-linking poly­(ethylene glycol) diacrylate (10 PEGDA) (Figure 1f). When the stiffnesses of HEA/BIS ELMs (63.72 ± 1.44 kPa) and PEGDA ELMs (62.36 ± 2.67 kPa) are similar, they show no significant difference in *E. coli* release (*P* > 0.05) (Figure 1f).

### 2.2 Microorganism Growth within ELMs Resulting in the Release of Microorganisms

Colony expansion induces the fracture of encapsulating matrices, resulting in cell release (Figure 2a). To investigate this proposed mechanism, we synthesized ELMs loaded with one colony-forming unit (*E. coli*). As the relatively stiff *E. coli* (with an elastic modulus > 1 MPa)^50^ multiply within the lower elastic modulus hydrogel matrix (7.52 ± 0.66 kPa), a colony is formed (day 1 to day 5, Figure 2b). Further proliferation increases the colony volume until it induces an observable fracture, resulting in cell release (day 6, Figure 2b). In this case, the shape of the fracture can be seen by the plane of fluorescent cells that remain within the fracture. This plane terminates at the ELM surface. For all ELMs, cell release occurs only after hydrogel fracture (Figure 2c), confirming the necessity of a fracture to achieve cell release. As is typical for both biological growth processes and failure processes, the exact time to fracture is somewhat stochastic. The fracture emanating from a growing colony may be similar to cavitation-induced failure in polymer networks.^51^ As such, fracture likely depends on the exact distance of the growing colony from the free surface.

**Figure 2:**
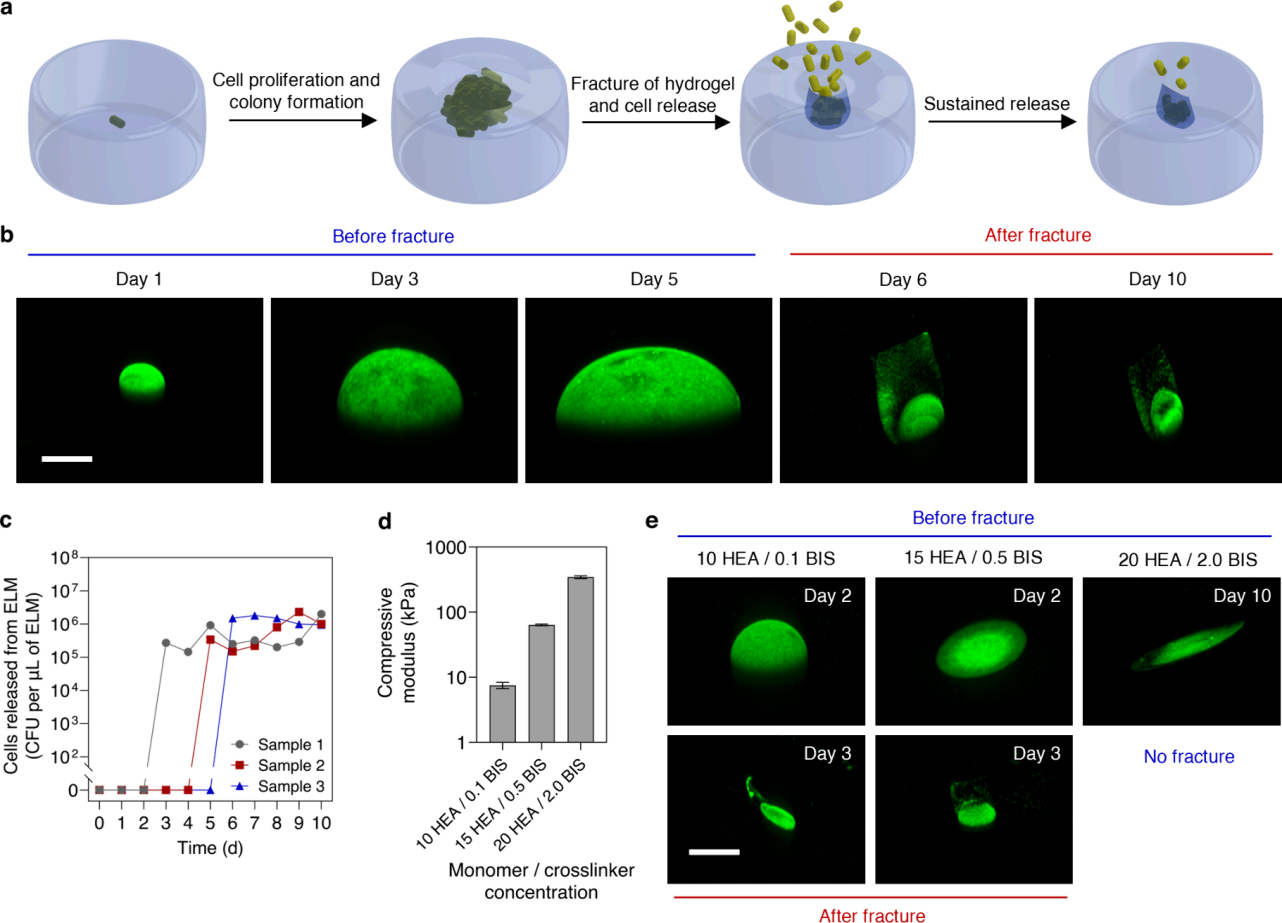
Mechanism for sustained release of microorganisms from ELMs. (a) Schematic illustrating the driving mechanism of cell release from ELMs. A single cell encapsulated within an ELM proliferates and forms a colony, which expands until it induces fracture and releases cells. (b) Confocal microscopy z-stack images showing colony enlargement and fracture in ELMs. Scale bar, 200 μm. The green fluorescence represents bacteria/bacterial colony, the black area represents the encapsulating hydrogel matrix, and image day 1 represents 24 h of incubation. (c) Cell release from ELMs as a function of time. ELMs in panels b and c were 1 mm thick, loaded with single *E. coli*, and prepared with a hydrogel formulation of 10 HEA/0.1 BIS. (d) Compressive modulus of hydrogels with different monomer/cross-linker (HEA/BIS) concentrations. (e) Microscopy z-stack images showing colony growth and fracture in ELMs with different stiffnesses (10 HEA/0.1 BIS, 15 HEA/0.5 BIS, and 20 HEA/2.0 BIS). Scale bar, 200 μm. ELMs in panel e were 0.5 mm thick and loaded with a single *E. coli*. Data in panel d are presented as mean ± standard deviations (*n* = 3).

Although the growth of cells within ELMs is necessary for inducing fracture and releasing cells, cell growth does not always result in fracture or cell release. The ability of the colony to induce fracture depends on the mechanical properties of the encapsulating matrices. We varied the stiffnesses of the hydrogels by varying their monomer (HEA) and cross-linker (BIS) ratios and observed the possibility of ELM fracture and cell release. Increasing the monomer/cross-linker concentrations from 10 HEA/0.1 BIS to 15 HEA/0.5 BIS and 20 HEA/2.0 BIS increases the compression modulus of the hydrogel from 7.52 ± 0.66 kPa to 63.72 ± 1.44 kPa and 345.16 ± 14.29 kPa, respectively (Figure 2d and Figure S3). At all stiffnesses, bacterial proliferation leads to colony formation and expansion within ELMs (Figure 2e). However, the stiffness of the hydrogels dictates the size and morphology of the colonies, as has been seen in other ELMs.^40,49^ The volume of the colony in low-stiffness ELMs is significantly larger than those of the medium-stiffness ELMs and high-stiffness ELMs (Figure S4a). In low-stiffness ELMs, the colony morphology is spherical, while in high-stiffness ELMs the colony morphology is plate-like. Likely, this change in morphology minimizes the elastic strain energy of the growing inclusion.^52^ The colony in the high-stiffness hydrogels is unable to cause fracture during the entire period. Therefore, no cell release is observed (Figure S4b). This suggests that the high stiffness of the hydrogel restricts the ability of the colony to reach a volume that can induce hydrogel fracture. This result also demonstrates that cell release can be tuned by varying the properties of the encapsulating matrices.

The volume of the encapsulating matrix plays a major role in allowing the fracture and subsequent cell release. Colonies encapsulated within a smaller volume of low-stiffness hydrogel have a better ability to induce hydrogel fracture compared to a larger volume of the same encapsulating matrix. We synthesized single-cell ELMs with different thicknesses (0.5, 1, and 2 mm) and observed whether the colony expansion induced hydrogel fracture. At all thicknesses, bacterial growth leads to colony formation and expansion within ELMs (Figure S5a). However, only 0.5 and 1 mm thick ELMs undergo fracture with colony expansion. Moreover, 1 mm thick ELMs require a significantly larger colony to induce fracture compared to 0.5 mm thick ELMs (Figure S5b). Hence, 1 mm thick ELMs demonstrate delayed fracture and delayed cell release. The hydrogel fracture in both 0.5 mm and 1 mm thick ELMs allows cell release in a sustained manner (Figure S5c). On the other hand, colonies in 2 mm thick ELMs are unable to induce fracture throughout the entire experiment (10 days). The larger hydrogel is better able to accommodate the strain caused by growing inclusion (colony) without failure. Critically, due to the absence of fracture, these ELMs do not release cells (Figure S5c), highlighting the importance of fracture to achieve cell release. These model ELMs with only a single colony help in understanding the mechanism of cell release from ELMs, but the number of released cells is somewhat difficult to control (Figures S4b and S5c).

Hydrogel degradation and the hydrogel mesh size did not contribute to this release mechanism. To confirm the inhibition of microorganism escape through the hydrogel network, we estimated the mesh size of the hydrogels. The mesh sizes of the hydrogels are 9.73 nm for 10 HEA/0.1 BIS, 3.42 nm for 15 HEA/0.5 BIS, 2.74 nm for 20 HEA/2.0 BIS, and 1.47 nm for 10 PEGDA. These mesh sizes are much smaller than the size of *E. coli*,^50^ confirming that the microorganisms cannot escape through the hydrogel network. To verify that these hydrogels do not degrade over time or due to bacterial activity, we incubated the hydrogels (10 HEA/0.1 BIS, 15 HEA/0.5 BIS, and 20 HEA/2.0 BIS) with *E. coli* in culture conditions (37 °C, 200 rpm) for 100 days and measured the changes in mass and mechanical properties of the hydrogels. In each condition, the materials do not significantly change in dry mass or compressive modulus (*P* > 0.05) (Figure S6), confirming that these HEA/BIS hydrogels do not degrade under the experimental conditions. These data further corroborate that the microorganism release is due to fracture.

### 2.3 Hydrogel Stiffness Controlling Microorganism Release

The stiffness of the encapsulating hydrogel matrix controls cell proliferation, colony formation, and cell release from the ELMs with many embedded cells. To increase the number of cells being released, we first increased the cell loading to 1 × 10^4^ cells per μL of ELM. The presence of a higher number of cells within ELMs increases the number of colonies and, likely, the number of fractures. In turn, cell release is increased as compared with the ELMs with a single colony. We synthesized hydrogels with the same cell loading (1 × 10^4^ cells per μL of ELM) but different stiffnesses by varying their formulations (10 HEA/0.1 BIS (low-stiffness), 15 HEA/0.5 BIS (medium-stiffness), and 20 HEA/2.0 BIS (high-stiffness)) and quantified their cell release. To determine if the monomers and cross-linkers used during the preparation of hydrogels produced any cytotoxic effect, we performed toxicity tests. There is no significant difference in cell viability when they are exposed to different concentrations of monomer and cross-linker (*P* > 0.05) (Figure S7a). Photo-cross-linking under these conditions also does not significantly reduce the cell viability for all formulations at these cell loadings (*P* > 0.05) (Figure S7b). Like the single-colony ELMs, the colony volume in these ELMs increases with time but decreases with hydrogel stiffness (Figure 3a). Low-stiffness ELMs have a significantly higher colony volume compared to both medium-stiffness (*P* < 0.0001) and high-stiffness ELMs (*P* < 0.0001) (Figure 3b). The number of viable cells present in the ELMs also increased with time. The number of cells present at day 10 within low-stiffness ELMs ((2.39 ± 0.18) × 10^8^ CFUs per μL of ELM) is significantly higher than those of both medium-stiffness ((7.45 ± 0.26) × 10^7^ CFUs per μL of ELM, *P* < 0.0001) and high-stiffness ELMs ((4.03 ± 0.18) × 10^7^ CFUs per μL of ELM, *P* < 0.0001) (Figure 3c). For all stiffnesses, the ELMs demonstrate release after 1 day, and within 3 days, the release reached a steady state (zero-order release kinetics, Figure 3d). This steady-state release remained for the duration of the experiment, which we ended arbitrarily after 10 (Figure 3d) and 100 days (Figure 3e). The increase in the cell release observed during the first 3 days could be attributed to new fractures occurring during that time, whereas the steady state observed after 3 days could indicate the lack of new fractures. During the steady state, the number of cells being released is proportional to the number of cells present within the ELMs and is primarily controlled by the doubling time of the microorganism. On day 10, the low-stiffness, medium-stiffness, and high-stiffness ELMs released (2.24 ± 0.06) × 10^8^, (1.85 ± 0.08) × 10^7^, and (3.14 ± 0.16) × 10^6^ CFUs per μL of ELM, respectively (Figure 3d). The low-stiffness ELMs release significantly more cells than both medium-stiffness (*P* < 0.0001) and high-stiffness ELMs (*P* < 0.0001), and the medium-stiffness ELMs release significantly more cells than high-stiffness ELMs (*P* < 0.01).

**Figure 3:**
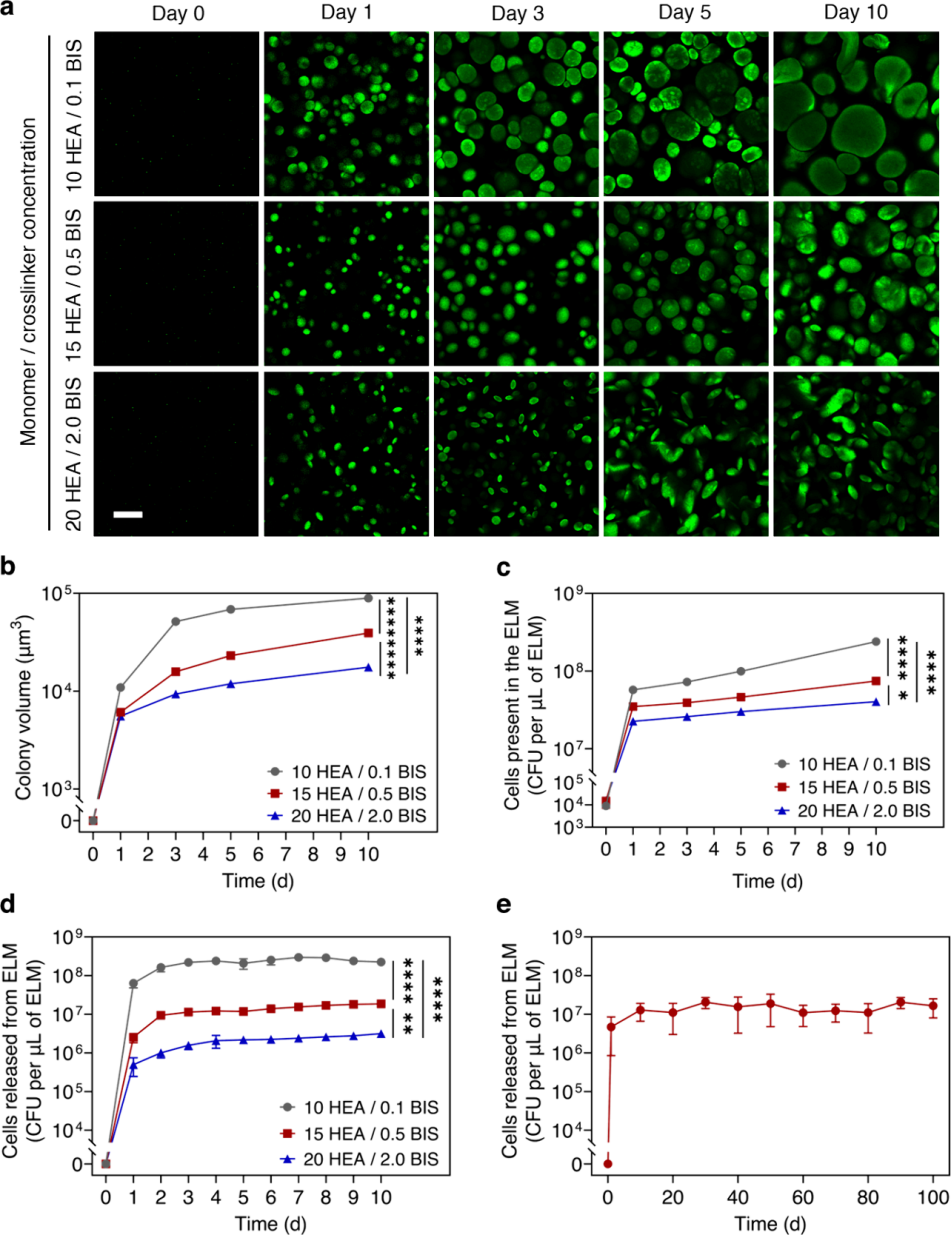
Hydrogel stiffness controls microorganism release. (a) Confocal microscopy images showing the differences in colony morphologies as the hydrogel stiffness is varied. Scale bar, 100 μm. (b) Colony volumes as a function of time for ELMs with different stiffnesses. (c) Number of cells present within ELMs as a function of time for ELMs prepared with different hydrogel stiffnesses. (d) Cells released from ELMs as a function of time for ELMs prepared with different hydrogel stiffnesses. Hydrogel stiffnesses were varied by varying the hydrogel formulations (10 HEA/0.1 BIS (gray), 15 HEA/0.5 BIS (red), and 20 HEA/2.0 BIS (blue)). (e) Cell release as a function of time from 15 HEA/0.5 BIS ELMs for 100 days. All ELMs were prepared with a cell loading of 1 × 10^4^ cells per μL of ELM. All data in panel b are presented as mean ± standard error of means (*n* > 1000), and all data in panels c and d are presented as mean ± standard deviations (*n* = 3). Statistical analysis was performed by a one-way ANOVA with posthoc Tukey’s test, * *P* < 0.05, ** *P* < 0.01, and **** *P* < 0.0001.

The cells released from the ELMs can proliferate in the growth medium, but cell proliferation in the growth medium does not significantly change the measured number of released cells in the first 2 h. For quantifying cell release, we measured the number of cells present in the medium after 2 h of incubation. In this method, the viable cells released from the ELMs can continue to proliferate in the growth media. To better quantify the cells released as compared to cells proliferated in the media, we measured colony-forming units present in the media under two conditions (Figure S8a). Five day-grown ELMs were suspended in fresh LB media and incubated at 37 °C and 200 rpm. Then, in condition 1, the ELMs were removed after 30 min and the remaining medium was incubated in the same conditions for the next 90 min. Here, the cells released from the ELMs in the first 30 min can further proliferate within the medium for the next 90 min. The number of cells present in the medium at 30 min did not increase significantly in the next 90 min (Figure S8b). On the other hand, in condition 2, the ELMs remained in the medium for the entire 2 h; hence, the ELMs release cells for the entire 2 h. The number of cells present in the medium at 30 min increased significantly in the next 90 min for all formulations (*P* < 0.0001 for low-stiffness ELMs, *P* < 0.01 for medium-stiffness ELMs, and *P* < 0.001 for high-stiffness ELMs) (Figure S8c). These data collectively support the idea that further proliferation of released cells does not significantly influence the number of cells measured at 2 h.

The proliferation of cells within ELMs is essential for the cell release. We compared the cell release from 5-day-grown ELMs in shaking conditions (37 °C, 200 rpm) with static conditions (37 °C). There was no significant difference between the number of cells released by the ELMs in shaking and static conditions (Figure S9). This suggests that the cell release is driven by the forces associated with cell proliferation and not collisions with the container. Moreover, ELMs only release cells in growth media such as LB and not in saline. However, when ELMs grown in LB are transferred to saline, lower cell release is observed, which reduces dramatically with time. To better quantify this residual cell release, 5-day-grown ELMs were incubated in LB medium and saline, and their cell release was compared at different time points (Figure S10a). During the first 30 min, there was no significant difference between the number of cells released in saline and LB medium (Figure S10b). Additionally, in saline, the number of cells released in the first 30 min did not increase significantly during the next 90 min (Figure S10c). In contrast, in LB medium, as discussed earlier, the cells released in the first 30 min significantly increased during the next 90 min. These data collectively suggest that the residual cell release plays a significant role in the first 30 min, whereas the forces associated with cell proliferation play a significant role during the next 90 min.

The ease of controlling probiotic release by just varying the stiffness of the encapsulating matrix demonstrates the potential of this technology for different types of diseases where the probiotic dose is crucial. These small (∼1 μL) ELMs release >10^8^ CFUs in 2 h, demonstrating the ability to release high doses of probiotics in a short period, which is necessary for therapeutic efficacy.^53,54^ Moreover, the release of high doses of probiotics is sustained for several days. Since repeated administration of a prescribed dose of viable probiotics for a long period can promote colonization,^33^ the ability of this approach to release high doses in a sustained manner for a prolonged time makes ELM a good candidate for achieving probiotic persistence/colonization.

### 2.4 Microorganism Release Being Tuned by Initial Cell Loading

Cell release can be varied by controlling the number of cells present in the ELMs. We quantified the cells released from ELMs synthesized with the same stiffness (15 HEA/0.5 BIS) but different initial cell loadings (10^0^ cells per μL of ELM, 10^4^ cells per μL of ELM, and 10^8^ cells per μL of ELM). The photopolymerization process does not significantly reduce the cell viability for ELMs with low- and medium-cell-loadings (10^0^ cells per μL of ELM and 10^4^ cells per μL of ELM) (*P* > 0.05), whereas it significantly reduces the cell viability for ELMs with high-cell-loading (10^8^ cells per μL of ELM) (*P* < 0.01) (Figure S11a). This decrease is likely due to the high monomer:medium ratio used while preparing high-cell-loading ELMs.^43^ However, increasing the cell loading still increases the number of viable cells (Figure S11b,c), which in turn increases the number of colonies present within the ELMs (Figure 4a). For ELMs with 10^8^ cells per μL of ELM (high*cell-loading), imaging individual colonies and quantifying colony volumes was not possible due to near-confluent growth (Figure 4a). We have previously demonstrated that cell proliferation within ELMs causes shape change^39,43^ accompanied by a change in topography,^39^ as the growing colonies deform the surface in a heterogeneous manner at the sub-millimeter scale. During confocal microscopy of high-cell-loading ELMs, the high cell density throughout the thickness of the hydrogels made it difficult to image cells/colonies located at the center. As a result, imaging was limited to cells and colonies near the surface. In the crevices on the surface, colonies are not present as there is no material in that region. Over 10 days of growth, all ELMs demonstrate an increase in the number of cells present within the ELMs (Figure 4b). Comparing ELMs with different cell loading after 10 days shows that high-cell-loading ELMs have a significantly higher number of cells ((1.6 ± 0.26) × 10^8^ CFUs per μL of ELM) than both medium-cell-loading ((7.45 ± 0.26) × 10^7^ CFUs per μL of ELM, *P* < 0.01) and low-cell-loading ELMs ((6.68 ± 2.54) × 10^6^ CFUs per μL of ELM, *P* < 0.001) (Figure 4b). Similarly, medium-cell-loading ELMs has significantly more cells than low-cell-loading ELMs (*P* < 0.05). For high and medium cell loading, the ELMs reached a steady-state release within 3 days and sustained this release for the remainder of the experiment (Figure 4c), whereas low-cell-loading ELMs demonstrate a delay in reaching a steady-state release (6 days). On day 10, the low-, medium-, and high-cell-loading ELMs released (8.93 ± 0.34) × 10^5^, (1.85 ± 0.08) × 10^7^, and (3.34 ± 0.05) × 10^8^ CFUs per μL of ELM, respectively. The high-cell-loading ELMs release significantly higher cells than both medium- (*P* < 0.0001) and low-cell-loading ELMs (*P* < 0.0001), and the medium-cell-loading ELMs release significantly more cells than low-cell-loading ELMs (*P* < 0.01). These data collectively suggest that cell release can be controlled by varying cells present within the ELMs.

**Figure 4:**
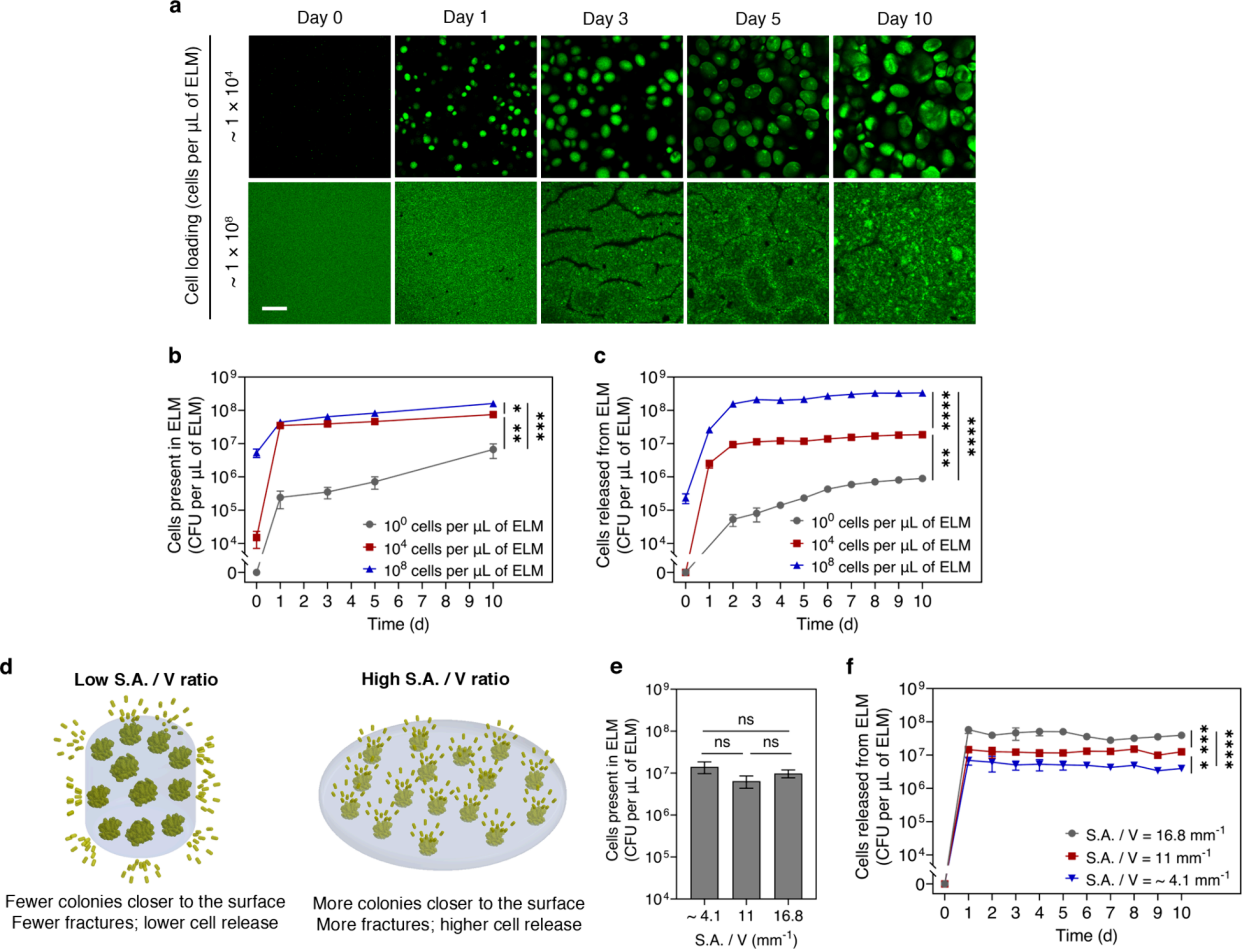
Controlling microorganism release by varying cell loading and shape of ELMs. (a) Confocal microscopy images showing cell proliferation and colonies within ELMs of different cell loadings. Scale bar, 100 μm. (b) Number of cells present within ELMs as a function of time for ELMs prepared with different cell loadings. (c) Cells released from ELMs as a function of time for ELMs prepared with different cell loadings. ELMs in panels b and c were prepared with 15 HEA/0.5 BIS formulation, and their cell loading was varied from 1 × 10^0^ cells per μL of ELM (gray) to 1 × 10^4^ cells per μL of ELM (red) and 1 × 10^8^ cells per μL of ELM (blue). The initial volume and surface-area-to-volume (S.A./*V*) ratios of all of the ELMs in panels a–c were the same. (d) Schematic showing influence of S.A./*V* ratio on cell release. ELMs with a high S.A./*V* ratio have more colonies closer to the surface, which induces more fracture, thereby releasing more cells than ELMs with a low S.A./*V* ratio. (e) Number of cells present within ELMs after 10 days of cell release for ELMs prepared with different S.A./*V* ratios (16.8, 11, and ∼4.1 mm^–1^). (f) Cell release as a function of time from ELMs with different S.A./*V* ratios (16.8 mm^–1^ (gray), 11 mm^–1^ (red), and ∼4.1 mm^–1^ (blue)). ELMs in panels e and f were prepared with a cell loading of 1 × 10^4^ cells per μL of ELM and 15 HEA/0.5 BIS formulation. All data are presented as the mean ± standard deviation (*n* = 3). Statistical analysis was performed by a one-way ANOVA with posthoc Tukey’s test. * *P* < 0.05, ** *P* < 0.01, *** *P* < 0.001, **** *P* < 0.0001, and not significant (ns) for *P* > 0.05.

### 2.5 Size and Shape of ELMs Controlling Microorganism Release

Not all colonies within ELMs induce hydrogel fracture and facilitate cell release. We hypothesized that the colonies that are closer to the surface have a higher probability of inducing rupture and, therefore, releasing cells (Figure 4d). We compared the cell release from ELMs with the same volume (2.48 ± 0.24 mm^3^) but different surface-area-to-volume (S.A./*V*) ratios (Figure 4d). Varying the S.A./*V* ratios does not significantly change the number of cells present in ELMs over 10 days of growth (Figure 4e and Figure S12). Moreover, ELMs with different S.A./*V* ratios all demonstrate zero-order release kinetics for the entire period (Figure 4f). However, increasing the S.A./*V* ratio from ∼4.1 to 11 and 16.8 mm^–1^ increases the cell release from (3.93 ± 0.66) × 10^6^ CFUs per μL of ELM to (1.25 ± 0.13) × 10^7^ CFUs per μL of ELM and (3.93 ± 0.41) × 10^7^ CFUs per μL of ELM, respectively (Figure 4f). ELMs with an S.A./*V* ratio of 16.8 mm^–1^ release significantly more cells than ELMs with an S.A./*V* ratio of 11 mm^–1^ (*P* < 0.001) and ∼4.1 mm^–1^ (*P* < 0.0001). Similarly, ELMs with an S.A./*V* ratio of 11 mm^–1^ also release significantly more cells than ELMs with an S.A./*V* ratio of ∼4.1 mm^–1^ (*P* < 0.05). These data suggest that colonies closer to the surface play a significant role in cell release. This phenomenon is somewhat expected due to potentially higher proliferation near the surface and the increased propensity for the growing colony near the surface to induce failure.^40,55^

ELM size also plays a role in controlling cell release. To test the controllability of cell release from ELMs of different sizes, we varied the volumes of the ELMs (2.47 and 105.5 mm^3^) without changing the S.A./*V* ratios (4.08 ± 0.02 mm^–1^). The smaller ELMs (2.47 mm^3^) demonstrate zero-order release kinetics after 1 day, whereas the larger ELMs (105.5 mm^3^) only reach zero-order release kinetics after 3 days (Figure S13). Moreover, as the initial volume of the ELMs increases from 2.47 to 105.5 mm^3^, the cell release from the ELMs reduces significantly from (3.93 ± 0.66) × 10^6^ CFUs per μL of ELM to (6 ± 1.91) × 10^5^ CFUs per μL of ELM (*P* < 0.05). The reason for reduced cell release for larger ELMs could be due to the inherent length scales associated with diffusion and consumption of nutrients, or the volume of media could also be disproportionally small for the volume of ELMs. Hence, this could induce competition between the cells present within the ELMs, resulting in further reduced cell release.

### 2.6 Other Microorganisms Being Released via the Same Mechanism

Since cell release is governed by mechanics and not chemical mechanisms, this mechanism can be extended to a wide range of probiotics. Moreover, the release of those probiotics can also be controlled using the same techniques, such as varying the stiffness of the encapsulating matrices and the initial cell loading. However, the rate of proliferation, size, and mechanical properties of the cells are controlled by the genetics of the organism, which leads to changes in the rate of release.^9,16,56,57^ We demonstrate the controlled release of probiotics that belong to a different bacterial phylum (*L. paracasei*, a Gram-positive bacterium) and a different kingdom (*S. cerevisiae*, a fungus). All of these organisms have higher stiffness relative to the synthesized hydrogels.^36,50,57^ We first synthesized ELMs loaded with *L. paracasei* (1 × 10^3^ cells per μL of ELM) with different stiffnesses by varying their formulations (10 HEA/0.1 BIS (low-stiffness), 15 HEA/0.5 BIS (medium-stiffness), and 20 HEA/2.0 BIS (high-stiffness)) and quantified their cell release (Figure 5a). The low-stiffness ELMs release significantly more cells than both medium-stiffness (*P* < 0.001) and high-stiffness ELMs (*P* < 0.001). Next, we quantified the cell release from medium-stiffness ELMs (15 HEA/0.5 BIS) loaded with different cell loadings (10^0^ cells per μL of ELM (low-cell-loading), 10^3^ cells per μL of ELM (medium-cell-loading), and 10^6^ cells per μL of ELM (high-cell-loading)) (Figure 5b). The high-cell-loading ELMs release significantly more cells than both medium- (*P* < 0.0001) and low-cell-loading ELMs (*P* < 0.0001). Similarly, comparing cell release from *S. cerevisiae*-loaded ELMs (1 × 10^3^ cells per μL of ELM) with different hydrogel stiffnesses (10 HEA/0.1 BIS (low-stiffness), 15 HEA/0.5 BIS (medium-stiffness), and 20 HEA/2.0 BIS (high-stiffness)) shows that low-stiffness ELMs release significantly more cells than both medium-stiffness (*P* < 0.0001) and high-stiffness ELMs (*P* < 0.0001) (Figure 5c). Also, comparing cell release from *S. cerevisiae*-loaded ELMs (15 HEA/0.5 BIS (medium-stiffness)) with different cell loadings (10^0^ cells per μL of ELM (low-cell-loading), 10^3^ cells per μL of ELM (medium-cell-loading), and 10^6^ cells per μL of ELM (high-cell-loading)) shows that high-cell-loading ELMs release significantly more cells than both medium- (*P* < 0.05) and low-cell-loading ELMs (*P* < 0.01) (Figure 5d). Compared with *E. coli*-loaded ELMs, a delay in cell release is observed in both *L. paracasei*-loaded ELMs and *S. cerevisiae*-loaded ELMs. Although both *L. paracasei*-loaded ELMs and *S. cerevisiae*-loaded ELMs demonstrate zero-order release kinetics, they require a longer time to reach steady-state release. The delay in both the start of release and the time taken to take steady-state could be attributed to the slow doubling time of *L. paracasei* and *S. cerevisiae* compared to *E. coli*.^9,16,56,57^

**Figure 5:**
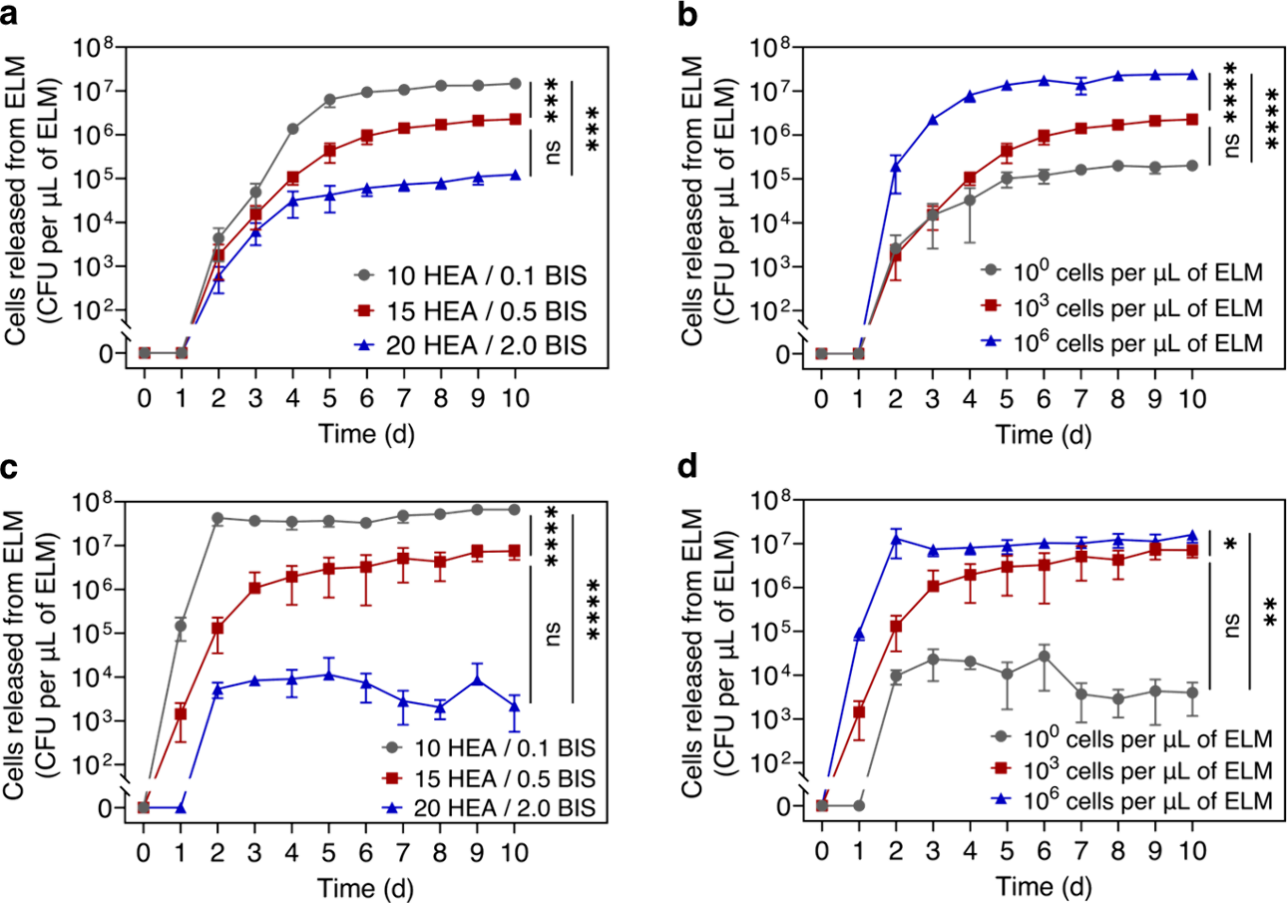
Controlled release of other microorganisms from ELMs. (a, b) Controlled release of *L. paracasei* by varying hydrogel stiffnesses and initial cell loading. (a) Cell release as a function of time from ELMs with different hydrogel stiffnesses. (b) Cell release as a function of time from ELMs with different cell loadings. (c, d) Controlled release of *S. cerevisiae* by varying hydrogel stiffnesses and initial cell loadings. (c) Cell release as a function of time from ELMs with different hydrogel stiffnesses. (d) Cell release as a function of time from ELMs with different cell loadings. All ELMs in panels a and c were prepared with a cell loading of 1 × 10^3^ cells per μL of ELM, and all ELMs in panels b and d were prepared with 15 HEA/0.5 BIS. All data are presented as mean ± standard deviations (*n* = 3). Statistical analysis was performed by a one-way ANOVA with posthoc Tukey’s test. * *P* < 0.05, ** *P* < 0.01, *** *P* < 0.001, **** *P* < 0.0001, and not significant (ns) for *P* > 0.05.

Using this fracture-based mechanism, different types of probiotics can be released from ELMs. In this work, we demonstrated controlled release of *E. coli* (ABU 83972), *L. paracasei*, and *S. cerevsiae* from ELMs. ABU 83972 is an *E. coli* strain (a Gram-negative bacterium) that offers superior protection against recurrent urinary tract infections (UTIs) compared to the current standard of treatment (antibiotic therapy),^16,58^ representing an alternative approach to antibiotics for preventing UTIs. ABU 83972 outcompetes uropathogenic *E. coli* and other common uropathogens but does not consistently persist or colonize the bladder.^9,16,34^ Hence, a bladder-resident ELM that releases ABU 83972 in a sustained manner could overcome this limitation, thereby conferring a potential alternative for treating UTIs and recurrent UTIs. Several probiotics have persistence or colonization limitations, which can be overcome by this approach. For example, *L. paracasei* is a Gram-positive bacterium that has been used as probiotics for treating UTIs, bacterial vaginosis, and fungal infections.^12^ Similarly, *S. boulardii* and *S. cerevisiae* are probiotics that belong to the fungal kingdom and have shown potential in treating diarrhea, colitis, bacterial vaginosis, and candida infections.^59^ These probiotics also have persistence limitations;^60,61^ hence, using a material that releases these probiotics in a sustained manner could overcome such limitations.

The release mechanism demonstrated above may be applicable to release in the GI tract. For example, *L. paracasei* is a GI tract probiotic. This probiotic can survive in the GI tract, but it typically does not persist.^61^ The *L. paracasei*-loaded ELMs release after exposure to simulated gastric conditions. ELMs (15 HEA/0.5 BIS; 1 × 10^6^ *L. paracasei* cells per μL of ELM) were exposed to simulated gastric fluid (SGF) (pH 2.5) for 20 and 40 min. There is no significant difference in the number of cells released from ELMs exposed to SGF for 0, 20, and 40 min (Figure S14), confirming that the ELMs release *L. paracasei* after exposure to harsh gastric conditions. The exposure times were chosen based on the gastric emptying time in young healthy adults, which is 15–45 min (both fasted and fed states).^62^ These results demonstrate the potential applicability of this release mechanism for future delivery devices in the gut.

In this work, we show controlled release from ELMs as materials. Here we use the term ELM to describe engineered composites of both living and nonliving components. We note that the organisms are not engineered but are selected for their probiotic properties. The ELM is engineered to control the release. We did not explicitly study the fracture mechanics of the hydrogel. A suitable future study to correlate fracture energy of hydrogels to release might quantify the energy release rate of cavitation in the hydrogel.^51^ In order to use controlled release in a medical device, substantial further engineering would be required. For example, this study demonstrates release from relatively small ELMs. Limitations in nutrient diffusion may affect the release of probiotics as the ELM is scaled in size.^40,55^ As a result, not all medical device form factors may be accessible. We plan to understand the nutrient flow and growth mechanics in thick ELMs in the future to overcome this limitation. Furthermore, although we demonstrate a mechanism that enables release for at least 100 days, this work does not demonstrate probiotic persistence at a target site. To achieve persistence, ELMs capable of sustained release should be retained at the target site. Hence, ELMs should be redesigned based on the application and target site to allow retention. In many applications, degradation of the matrix may be desirable. However, degradation will also lead to changes in the mechanical properties. Therefore, we expect changes in the probiotic release during degradation. Finally, there may be opportunities to engineer the probiotic to further enhance retention or functional benefits of the probiotic.

## 3 Conclusions

We report a simple yet powerful method to release microorganisms in a sustained and controlled manner by using ELMs. In these ELMs, the encapsulating hydrogel matrix maintains the viability of the microorganisms and allows for microbial proliferation into colonies. Further proliferation expands the colonies and induces fracture, resulting in a microbial release. Using single-cell ELMs, we demonstrate that the cell location, along with the stiffness and volume of the encapsulating matrix, dictates hydrogel fracture. We control cell release by varying the initial cell loading and encapsulating matrix properties such as stiffness, size, and shape. Since the driving mechanism for this approach is mechanical, this mechanism can be extended to several probiotics and synthetic polymers. Hence, we demonstrate sustained release of *E. coli* (a Gram-negative bacterium), *L. paracasei* (a Gram-positive bacterium), and *S. cerevisiae* (a fungus) for up to 10 days. We also show the release of microorganisms from two different classes of acrylic hydrogels. Although probiotics demonstrate great potential in treating complex diseases, a lack of persistence or colonization reduces the effectiveness of these therapies. This ELM-based technique may allow for the persistent delivery of a wide variety of probiotics at a target site and aid colonization, thereby improving the treatment efficacy. Furthermore, the simple fabrication technique and release mechanism may enable large-scale fabrication and translation of this technology to practical applications.

## 4 Experimental Section

### 4.1 Materials

2-Hydroxyethyl acrylate (HEA), *N*,*N*′-methylenebis­(acrylamide) (BIS), polyethylene glycol diacrylate (PEGDA, MW = 700 g mol^–1^), lithium phenyl-2,4,6-trimethylbenzoylphosphinate (LAP), sodium chloride (NaCl), pepsin, and hydrochloric acid (HCl) were purchased from Sigma-Aldrich. The ABU 83972 strain of *E. coli* was obtained from Subash Lab,^16,63^ the *L. paracasei* D3-5 strain was obtained from Yadav Lab,^64^ and *S. cerevisiae* (Active Dry) was obtained from Fleischmann’s Yeast. Powdered forms of Luria–Bertani (LB) Lennox broth, De Man, Rogosa, and Sharpe (MRS) broth, Yeast Peptone Dextrose (YPD) broth, and agar were purchased from BD Difco.

LB broth, MRS broth, and YPD broth were prepared with dH_2_O. All media–agar plates (LB-agar, MRS-agar, YPD-agar) were prepared with dH_2_O and 1.5 wt % agar. All growth media and PBS were sterilized by being autoclaved at 120 °C for 20 min and then stored at room temperature. Simulated gastric fluid (SGF)^65^ was prepared by dissolving 2 g of NaCl and 3.2 mg of pepsin in 500 mL of dH_2_O. Next, 7 mL of HCl was added, and the volume was adjusted to 1 L with additional dH_2_O. The pH was then adjusted to 2.5, and the solution was filter sterilized (poly­(ether sulfone), 0.2 μm).

### 4.2 Cell Culture

#### 4.2.1 Bacterial Culture

The ABU 83972 strain of *E. coli* was used for Gram-negative bacteria release studies. Bacterial cultures were grown in LB broth. Initially, *E. coli* from the glycerol stock was streaked onto LB-agar plates and incubated at 37 °C for 24 h. A single colony from the LB-agar plate was added to 25 mL of LB broth and incubated at 37 °C at 200 rpm (*n* = 3). After 24 h of incubation, the cultures were centrifuged at 4000 rpm for 10 min at room temperature, and the supernatants were removed. The bacterial cultures were then washed thrice with 25 mL of PBS to remove the culture media. The bacterial suspension was then diluted and adjusted to an optical density of 3.0 at 600 nm using a UV–vis spectrophotometer (Genesys 40, Thermo Scientific). To confirm the viable cell count, the solution was further diluted and plated on LB-agar plates. These plates were then incubated at 37 °C for 24 h, and the CFUs were counted. The bacterial suspensions had ∼2 × 10^5^ CFU μL^–1^, which was then further diluted or concentrated for preparing ELMs.

For microscopy, ABU 83972 which expresses red fluorescence protein (RFP) was used. All of the culture and growth were performed using the same procedure mentioned above, except, LB-amp broth and LB-amp agar plates (50 μg of ampicillin per mL of media) were used instead of LB broth and LB-agar plates.

*L. paracasei* was used for Gram-positive bacterial release studies. Initially, *L. paracasei* from the glycerol stock was streaked onto MRS-agar plates. These plates were then incubated at 37 °C with 5% CO_2_ for 48 h. A single colony from the MRS-agar plate was added to 25 mL of MRS broth and incubated at 37 °C with 5% CO_2_ (*n* = 3). After 24 h of incubation, the cultures were centrifuged at 4000 rpm for 10 min at room temperature and the supernatants were removed. The cells were then washed three times with PBS. The bacterial suspension was then diluted and the optical density adjusted at 600 nm to 1.7 using a UV–vis spectrophotometer. The solution was then further diluted and plated on the MRS-agar plates. These plates were then incubated at 37 °C and 5% CO_2_ for 48 h, and the CFUs were counted. The bacterial suspensions had ∼1 × 10^5^ cells μL^–1^, which was then further diluted or concentrated for preparing ELMs.

#### 4.2.2 Yeast Culture

*S. cerevisiae* was used for yeast release studies. Initially, 10 mg of dry *S. cerevisiae* (Fleischmann’s Yeast) was dissolved in 1 mL of sterile dH_2_O, and the solution was streaked onto YPD-agar plates. These plates were then incubated at 30 °C for 48 h. A single colony from the YPD-agar plate was added to 25 mL of YPD broth and incubated at 30 °C under aerobic conditions with constant shaking at 200 rpm (*n* = 3). After 24 h of incubation, the cultures were centrifuged at 4000 rpm for 10 min at room temperature, and the supernatants were removed. The cells were then washed three times with PBS. The yeast suspension was then diluted, and the optical density at 600 nm was measured using a UV–vis spectrophotometer. For all replicates, the dilutions were adjusted to obtain an optical density of 1.4 at 600 nm. The solution was then further diluted and plated on YPD-agar plates. These plates were then incubated at 30 °C for 48 h, and the CFUs were counted. The yeast suspensions had ∼1 × 10^5^ cells μL^–1^, which was then further diluted or concentrated for preparing ELMs.

### 4.3 Preparation of ELMs

#### 4.3.1 ELMs Loaded with *E. coli*

The ELMs were prepared by free radical polymerization of HEA (monomer) and BIS (cross-linker). LAP was used as a photoinitiator for this polymerization. Stock solutions of BIS (0.02 and 0.04 g mL^–1^) and LAP (0.02 g mL^–1^) were prepared in dH_2_O. All chemicals and stock solutions were filter sterilized (poly­(ether sulfone), 0.2 μm) before ELM preparation. ELMs with varying stiffnesses were prepared with a cell loading of 1 × 10^4^ cells μL^–1^. The ELMs with low-stiffness hydrogel (10 HEA/0.1 BIS), medium-stiffness hydrogel (15 HEA/0.5 BIS), and stiff hydrogel (20 HEA/2.0 BIS) were prepared using 10 wt % HEA and 0.1 wt % BIS; 15 wt % HEA and 0.5 wt % BIS; and 20 wt % HEA and 2.0 wt % BIS, respectively. The concentration of each component denotes the percentage by weight in the entire pregel solution, including cells. All ELMs were prepared with 0.04 wt % LAP. The remaining fraction of the pregel solution was filled with an equivalent mass of LB media. ELMs with varying cell loadings were prepared with medium-stiffness hydrogels (15 HEA/0.5 BIS). of 1 × 10^4^ cells μL^–1^. The ELMs with low, medium, and high cell loading were prepared with 1 × 10^0^, 1 × 10^4^, and 1 × 10^8^ cells μL^–1^, respectively. In these formulations, as the biomass was increased, the LB-medium volume was reduced, such that the total pregel solution amounted to 100 wt %. All of the solutions were then vortexed for ∼5 s. After preparation, these pregel solutions were filled into polyethylene tubing (PE50, Braintree Scientific) (Method #1) and exposed to UV irradiation (UVP cross-linker CL-3000, Analytik Jena) of 365 nm at an intensity of 1.2 mW cm^–2^ for 2 min to polymerize. The polymerized ELMs were then dispensed from the tubing with a sterile 27G needle, trimmed to a 4 mm length with razor blades, and washed thrice with PBS to remove unpolymerized monomer residues. The volume of the resulting ELMs was ∼1 μL.

For preparing ELMs with different volumes or different surface-area-to-volume ratios (S.A./*V*), medium cell loading (1 × 10^4^ cells μL^–1^) and medium-stiffness hydrogel formulation (15 HEA/0.5 BIS) were used. These ELMs were prepared by polymerizing the pregel solutions in a mold (Method #2). Molds were prepared by using two glass slides (75 × 50 × 1 mm^3^) treated with a water-repellent spray (Rain-X Original), separated by a spacer. The thickness of the ELMs was defined as the thickness of the spacers. Different thicknesses of spacers that were used include 0.125 0.2, 0.25, 0.5, 1, 1.4, and 2 mm. Upon the preparation of pregel solutions, the solutions were filled into molds and exposed to UV irradiation of 365 nm at an intensity of 1.2 mW cm^–2^ for 2 min to polymerize. The molds were flipped every 30 s during polymerization. After polymerization, the samples were removed from the glass slides, cut to the required diameter using biopsy punches, and washed three times with PBS to remove unpolymerized monomer residues. Different diameters of biopsy punches used were 2.5, 4, 5, and 12 mm.

For preparing ELMs with a single colony, a cell loading of 1 × 10^0^ cells μL^–1^ was used to prepare the pregel solutions. The ELMs were prepared using molds by following the same procedure (Method #2). The prepared ELMs were incubated at 37 °C in aerobic conditions with constant shaking at 200 rpm for 24 h. The 1-day-grown ELMs with no colony, > 1 colony, or any colonies near the surface of the hydrogels were not used for further experimentation. For growth, all ELMs were incubated in 10 mL of LB-amp medium and the medium was refreshed every 24 h for 10 days.

PEGDA ELMs were prepared by the free radical polymerization of PEGDA (monomer) in aqueous solution. LAP was used as a photoinitiator for this polymerization. For preparing the pregel solution, a medium cell loading (1 × 10^4^ cells μL^–1^) of *E. coli* and 10 wt % PEGDA (monomer) were used. The pregel solutions were then polymerized using the same procedure (Method #1).

Cell-free hydrogel samples used for mechanical characterization were also prepared using this approach (Method #2), but no cells were added to the pregel solution.

#### 4.3.2 *L. paracasei*-Loaded ELMs and *S. cerevisiae*-Loaded ELMs

The ELMs were prepared using the same procedure (Method #1), except ELMs with varying stiffnesses were prepared with a cell loading of 1 × 10^3^ cells μL^–1^; ELMs with varying cell loadings were prepared with a cell loading of 1 × 10^0^, 1 × 10^3^, and 1 × 10^6^ cells μL^–1^. During preparation, MRS medium was used for *L. paracasei*-loaded ELMs, and YPD medium was used for *S. cerevisiae*-loaded ELMs.

### 4.4 Imaging and Quantification of *E. coli* Colony Growth within ELMs

ELMs loaded with RFP expressing ABU 83972 (*E. coli*) were imaged using a confocal microscope (SP8, Leica Microsystems). The ELMs were exposed to a laser wavelength of 554 nm and power of 2%, and the detection wavelength was 540 – 650 nm and shown with a green pseudocolor. Images were acquired with a resolution of 512 × 512 pixels by using a 10× lens. z-stacks of 500 μm were taken in steps of 0.2 μm.

The z-stack images were processed using Imaris software (Version 10.1, Oxford Instruments). The z-stack images were converted to 3D surfaces using the Imaris surface tool. The surfaces were generated with a smooth function set to 2 μm, background threshold set to 10 μm, and minimum surface voxel limit set to 10. The volumes of all bacterial colonies within the field of view were calculated.

### 4.5 Quantification of Bacteria/Yeast Release from ELMs

To quantify the release of *E. coli* from ELMs, the samples were placed in a 50 mL centrifuge tube with 10 mL of LB medium and incubated at 37 °C in aerobic conditions with constant shaking at 200 rpm for 24 h. After 2 h of incubation, an aliquot of the medium was diluted (in 10-fold dilutions, as necessary) and plated on LB-agar plates using an automatic plater (easySpiral, Interscience). These plates were then incubated at 37 °C overnight, and the CFUs were counted using an automatic colony counter (Scan 300, Interscience). Every 24 h, the ELMs were removed from the LB media and washed thrice with PBS (each wash comprised vortexing the ELM with 10 mL of PBS at a medium speed for 20–30 s using a vortex mixer). The ELMs were then transferred to 10 mL of fresh LB media and incubated in the same conditions. After 2 h of incubation, the cell release was measured using the same procedure. The release of *E. coli* from the ELMs was measured for 10 days. For the long-term release study, the same procedure was followed to measure the *E. coli* release for 100 days.

To quantify the release of *L. paracasei* from ELMs, the samples were placed in 10 mL of MRS media and incubated at 37 °C with 5% CO_2_ and no shaking for 24 h. After 6 h of incubation, an aliquot of the medium was diluted and plated on MRS-agar plates using the automatic plater. These plates were then incubated at 37 °C with 5% CO_2_ for 48 h and the CFUs were counted using the automatic colony counter. Every 24 h, the ELMs were removed from the medium, washed three times with PBS, transferred to fresh medium, and incubated in the same conditions. After 6 h of incubation, the cell release was measured using the same procedure. The release of *L. paracasei* from the ELMs was measured for 10 days.

To quantify the release of *S. cerevisiae* from ELMs, the samples were placed in 10 mL of YPD medium and incubated at 30 °C under aerobic conditions with constant shaking at 200 rpm for 24 h. After 6 h of incubation, an aliquot of the medium was diluted and plated on YPD-agar plates using the Copacabana method. These plates were then incubated at 30 °C for 48 h, and the CFUs were counted manually. Every 24 h, the ELMs were removed from the medium, washed thrice with PBS, transferred to a fresh medium, and incubated in the same conditions. After 6 h of incubation, the cell release was measured using the same procedure. The release of *S. cerevisiae* from the ELMs was measured for 10 days.

### 4.6 Swelling Studies

Freshly prepared 1 mm thick hydrogels (10 HEA/0.1 BIS, 15 HEA/0.5 BIS, 20 HEA/2.0 BIS, and 10 PEGDA) were washed with PBS and punched into 6 mm diameter discs. Immediately after washing, the front and top views of the hydrogels were photographed by using a digital single-lens reflex camera (EOS Rebel T7i, Canon). The images were analyzed using ImageJ to calculate the volumes (*v*_r_). For swollen volume measurements (*v*_s_), fresh hydrogels were immersed in PBS for 24 h to reach swelling equilibrium and then imaged and analyzed. For dry volume measurements (*v*_d_), both fresh hydrogels and swollen hydrogels were placed in a 70 °C oven for 24 h to ensure complete drying. The dried samples were then imaged and analyzed to calculate the volumes.

### 4.7 Mesh Size Estimation

The data obtained from the swelling studies were used to estimate the mesh sizes of hydrogels (10 HEA/0.1 BIS, 15 HEA/0.5 BIS, 20 HEA/2.0 BIS, and 10 PEGDA). The average molecular weights between cross-links (*M*_C_) were calculated using the Flory–Rehner equation modified by Peppas and Merrill,^66^

$$\frac{1}{M_{C}}=\frac{2}{M_{n}}-\frac{\{\mathrm{\nu ̅}\left[\ln\left(1-v_{2,s}\right)+v_{2,s}+\chi v_{2,s}^{2}\right]\}/V_{1}}{\left[{\left(\frac{v_{2,s}}{v_{2,s}}\right)}^{1/3}-\frac{1}{2}\frac{v_{2,s}}{v_{2,r}}\right]}$$

where *M*_*n*_ is the polymer average molecular weight before cross-linking; *V*_1_ is the molar volume of the water solvent (18 cm^3^ mol^–1^); *v*_2,s_ is the polymer volume fraction of the fully swollen hydrogel (*v*_2,s_ = *v*_s_/*v*_d_); *v*_2,r_ is the polymer volume fraction of the hydrogel immediately after cross-linking (*v*_2,r_ = *v*_r_/*v*_d_); *v̅* is the specific volume of the polymer (0.989 cm^3^ g^–1^ for HEA and 0.893 cm^3^ g^–1^ for PEGDA); and χ is the Flory–Huggins polymer–solvent interaction parameter (0 < χ > 0.5 for HEA–water^67^ and 0.4 for PEGDA–water^68^).

The root-mean-square end-to-end distance of the polymer chain in the unperturbed state (solvent-free) (*r̅*_0_^2^)^1/2^ was calculated as^68^

$${\left({{\mathrm{r̅}}_{0}}^{2}\right)}^{1/2}=l{C_{n}}^{1/2}\left(2\frac{M_{C}}{M_{r}}\right)$$

where *l* is the average bond length (0.154 nm for HEA and 0.146 nm for PEGDA^68^), *C*_*n*_ is the characteristic ratio of the polymer (typically 9.1 for HEA^69^ and 4.0 for PEGDA^68^), *M*_C_ is the average molecular weight between cross-links, and *M*_r_ is the molecular weight of the monomer.

The mesh size (ξ) of the hydrogel was finally calculated using the Canal and Peppas equation,^68,70^

$$\xi ={\left({{\mathrm{r̅}}_{0}}^{2}\right)}^{1/2}{v_{2,s}}^{-1/3}$$

where (*r̅*_0_^2^)^1/2^ is the root-mean-square end-to-end distance of the polymer chain in the unperturbed state (solvent-free) and *v*_2,s_ is the polymer volume fraction of the fully swollen hydrogel.

### 4.8 Degradation Studies

Freshly prepared 1 mm thick hydrogels (10 HEA/0.1 BIS, 15 HEA/0.5 BIS, and 20 HEA/2.0 BIS) were washed with PBS and punched into 6 mm diameter discs. The samples were then dried in a 70 °C oven for 24 h, and the dry masses were measured (Day 0). To quantify the degradability of hydrogels over time or due to bacterial activity, the hydrogels (10 HEA/0.1 BIS, 15 HEA/0.5 BIS, and 20 HEA/2.0 BIS) were placed in LB medium with *E. coli* (ABU 83972, 1× 10^3^ CFU/mL) and incubated at 37 °C and 200 rpm. Every 24 h, the hydrogels were removed from the LB medium, washed with PBS, transferred to fresh LB medium, and incubated in the same conditions. The same procedure was followed for 100 days. After 100 days, the samples were removed from LB medium, washed with PBS, and then immersed in 70% ethanol for 30 min to kill the bacteria. The samples were then swollen in dH_2_O for 24 h and then equilibrated in LB medium at 4 °C for 24 h. The samples were then dried in a 70 °C oven for 24 h, and the dry masses were measured (Day 100). Finally, the dry masses on Day 0 were compared to the dry masses on Day 100 to measure the degradation.

For mechanical characterization of Day 100 samples, the same procedure was followed on 12 mm diameter and 2 mm thick hydrogel samples (10 HEA/0.1 BIS, 15 HEA/0.5 BIS, and 20 HEA/2.0 BIS).

### 4.9 Mechanical Characterization

Cell-free hydrogel samples (12 mm in diameter and 2 mm in thickness) were equilibrated in LB medium at 4 °C for 24 h. A uniaxial compression test was performed at room temperature using a dynamic mechanical analyzer (RSA-G2, TA Instruments). The samples were loaded between the plates, and ∼30 mL of LB medium was added to the immersion system. The compression was then performed at a strain rate of 0.05 mm s^–1^ under immersion conditions. Strains between 0.2 and 5% were used to calculate Young’s modulus, as the stress–strain response in the region was linear.

### 4.10 Monomer/Cross-Linker Toxicity

The toxicities of the monomer and cross-linker were determined for *E. coli*, *L. paracasei*, and *S. cerevisiae*. To quantify the CFUs after exposure of each cell loading to each monomer/cross-linker concentration, all ELM formulations were prepared as described in the preparation of the ELM section, except that LAP was omitted to prevent gelation. Control solutions were prepared by replacing all monomers with LB media. After 2 min of exposure, an aliquot of the formulation was diluted (as necessary) and plated on LB-agar plates. These plates were incubated at 37 °C overnight and CFUs were counted.

### 4.11 Quantification of Bacteria Present within ELMs

ELMs were washed three times with PBS and placed in 3 mL of PBS for 30 min. ELMs were then homogenized using a hand-held homogenizer blade (Homogenizer 150, Fisherbrand) at high speed for ∼30 s (until no visible hydrogel fragments remained). The homogenizer blade was decontaminated with 70% EtOH and washed with sterile dH_2_O between samples. After homogenization, an aliquot of the solution was diluted (as necessary) and plated on LB-agar plates. These plates were then incubated at 37 °C overnight, and the CFUs were counted.

### 4.12 Quantification of Bacterial Proliferation from Released Cells

Five-day-grown ELMs were washed three times with PBS and incubated in 10 mL of fresh LB media at 37 °C and 200 rpm. In condition 1, the ELMs were removed after 30 min, and the remaining medium was incubated in the same conditions for the next 90 min. In condition 2, the ELMs remained in the medium for the entire 2 h. For both conditions, aliquots of the media at both 30 and 120 min were diluted (as necessary) and plated on LB-agar plates. These plates were then incubated at 37 °C overnight, and the CFUs were counted.

### 4.13 Tolerance Test in Simulated Gastric Fluid

Freshly prepared ELMs (15 HEA/0.5 BIS; 1 × 10^6^ *L. paracasei* cells per μL of ELM) were immersed in 10 mL of simulated gastric juice (SGF) for 0, 20, and 40 min (37 °C, 5% CO_2_). ELMs were then removed from SGF, placed in 10 mL of MRS medium, and incubated at 37 °C with 5% CO_2_ with no shaking. After 6 h of incubation, an aliquot of the medium was diluted and plated on MRS-agar plates. These plates were then incubated at 37 °C with 5% CO_2_ for 48 h, and the CFUs were counted. Every 24 h, the ELMs were removed from the medium, washed thrice with PBS, transferred to a fresh medium, and incubated in the same conditions. After 6 h of incubation on each day, the cell release was measured using the same procedure. The release was measured for 5 days.

### 4.14 Statistical Analysis

Statistical analysis was performed using GraphPad Prism (Version 10.2.3). Data are shown as the mean ± standard deviation. For all statistical tests, *P* < 0.05 was set for statistical significance. Single comparisons were performed using a two-tailed Student’s *t*-test (unpaired), and multiple comparisons were performed using a one-way analysis of variance (ANOVA) with a posthoc Tukey test.

## Supplementary Material



## Notes

Schematic showing steps involved in quantifying cell release and cell present within ELMs; hydrogel preparation stress–strain curves; influences of hydrogel stiffness, ELM size, bacterial activity, incubation conditions, cell proliferation, incubation conditions, incubation media, and size and shape of ELMs; cell viabilities; sustained cell release (PDF)
