# Supplementary material for: Controlled Release of Microorganisms from Engineered Living Materials
Source: ACS Appl Mater Interfaces. 2025 Jul 8;17(28):40326–39. doi: 10.1021/acsami.5c11155 (PMC12278213; doi:10.1021/acsami.5c11155)
Supplement: Supplementary file 1 [file am5c11155_si_001.pdf]

Supporting Information

Controlled release of microorganisms from engineered living materials

Manivannan Sivaperuman Kalairaj[1], *Iris George*[2], *Sasha M. George*[3], *Sofía E. Farfán*[1,4], *Yoo Jin Lee*[1], *Laura K. Rivera-Tarazona*[1], *Suitu Wang*[3], *Mustafa K. Abdelrahman*[3], *Seelay Tasmim*[1], *Asaf Dana*[1], *Philippe E. Zimmern*[5], *Sargurunathan Subashchandrabose*[2,*], *Taylor H. Ware*[1,3,*]

[1]Department of Biomedical Engineering, Texas A&M University, College Station, TX 77843, USA.

[2]Department of Veterinary Pathobiology, College of Veterinary Medicine and Biomedical Sciences, Texas A&M University, College Station, TX 77843, USA.

[3]Department of Materials Science and Engineering, Texas A&M University, College Station, TX 77843, USA.

[4]School of Engineering, Pontificia Universidad Católica de Chile, Santiago, 7820436, Chile.

[5]Department of Urology, The University of Texas Southwestern Medical Center, Dallas, TX 75390, USA.

*Corresponding Author Email: taylor.ware@tamu.edu, sarguru@cvm.tamu.edu

This document includes:

Figures S1 to S14

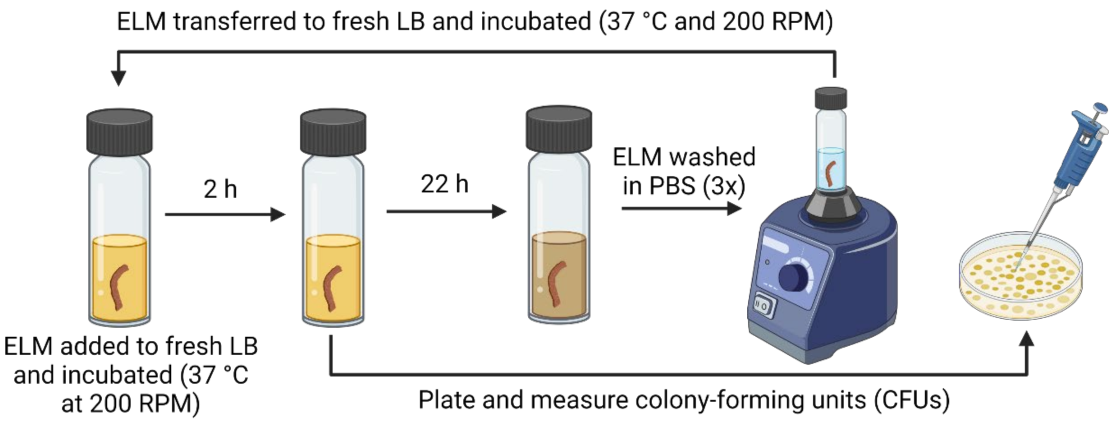

Figure S1. Schematic showing the steps involved in quantifying cell (*E. coli*) release from ELMs.

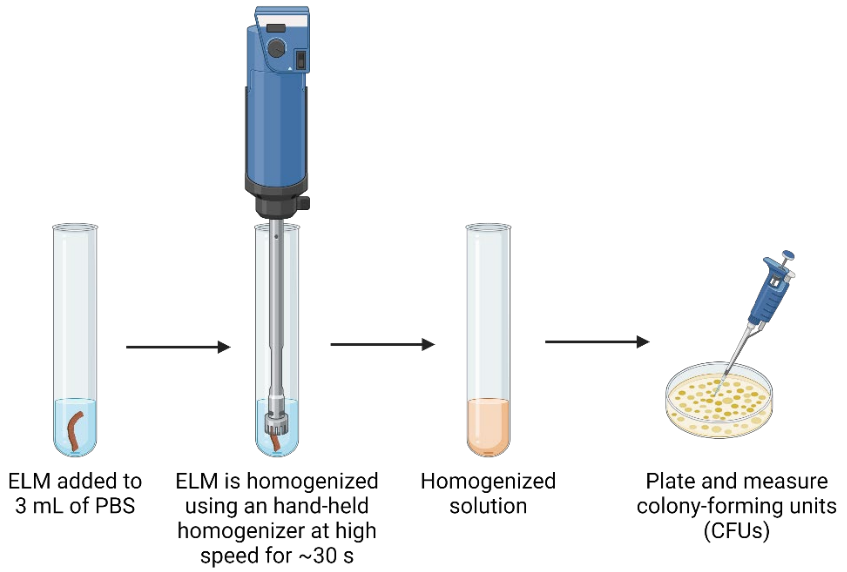

Figure S2. Schematic showing the steps involved in quantifying cells (*E. coli*) present within ELMs.

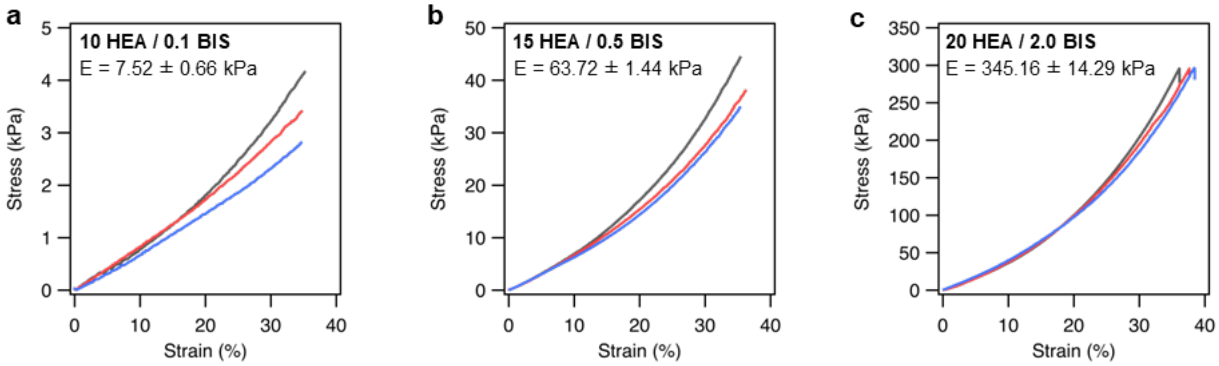

Figure S3. Stress-strain curves of HEA / BIS hydrogels (n = 3). **(a)** 10 HEA / 0.1 BIS, **(b)** 15 HEA/ 0.5 BIS, **(c)** 20 HEA / 2.0 BIS.

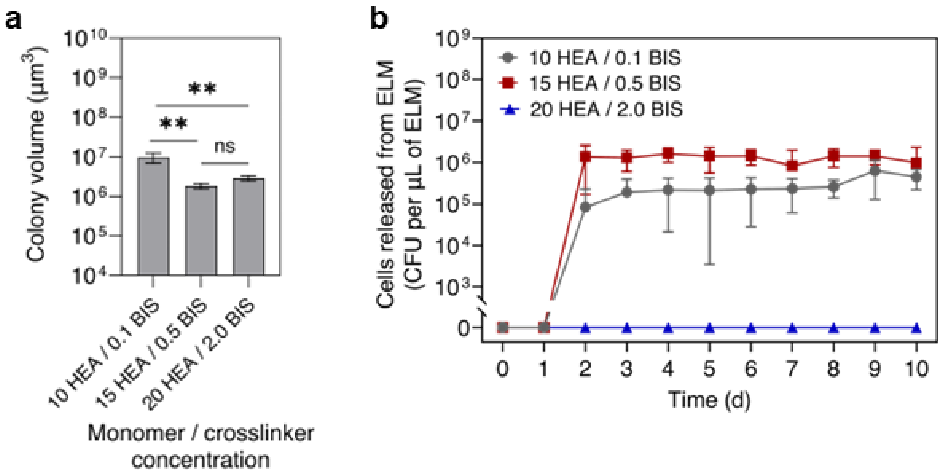

Figure S4. Influence of hydrogel stiffness on fracture and cell release. **(a)** Colony volume as a function of different hydrogel stiffnesses (10 HEA / 0.1 BIS, 15 HEA/ 0.5 BIS, and 20 HEA / 2.0 BIS). **(b)** Cell release as a function of time from ELMs with different stiffnesses. All ELMs were 0.5 mm thick and loaded with a single *E. coli*. All data are presented as mean ± standard deviation (n = 3). Statistical analysis was performed by a one-way ANOVA with post-hoc Tukey's test, ** $P < 0.01$, not significant (ns) for $P > 0.05$.

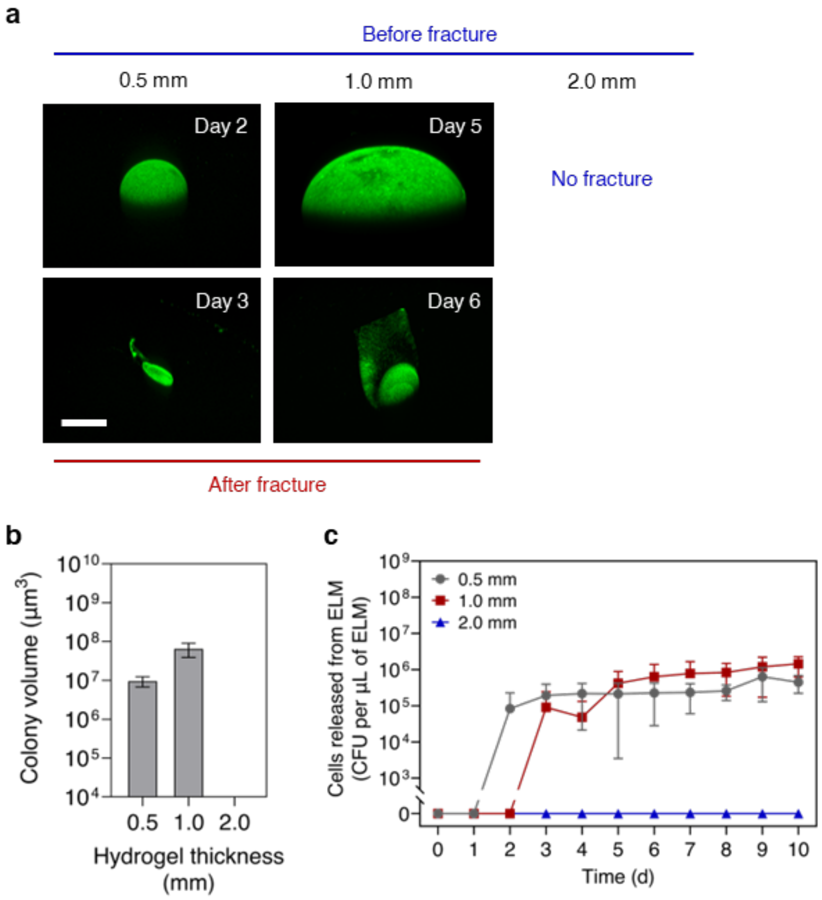

Figure S5. Influence of hydrogel thickness on fracture and cell release. **(a)** Confocal microscopy z-stack images showing colony growth and fracture in ELMs with different thicknesses. Scale bar, 200 μm. **(b)** Colony volume as a function of different hydrogel thicknesses. **(c)** Cell release as a function of time from ELMs with different thicknesses (0.5, 1, and 2 mm). All ELMs were prepared with low-stiffness hydrogel (10 HEA / 0.1 BIS) and loaded with a single *E. coli*. All data are presented as mean ± standard deviation (n = 3).

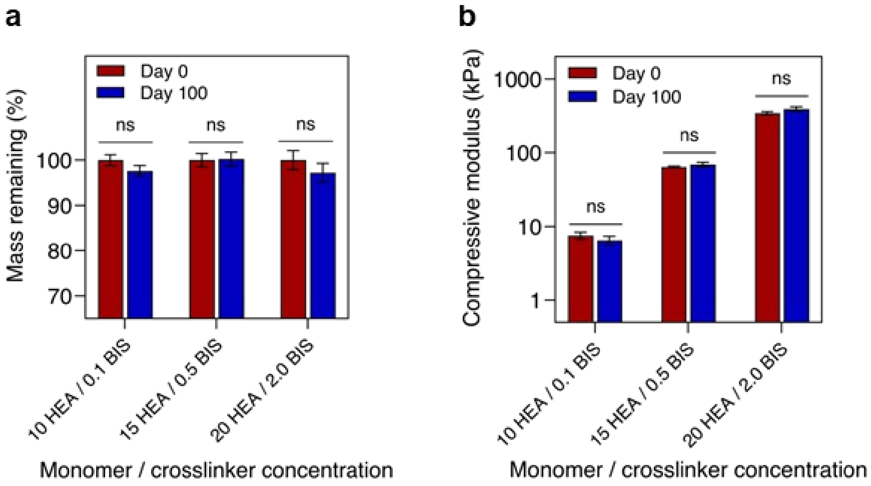

Figure S6. Influence of bacterial activity and incubation conditions on hydrogel degradation. **(a)** Mass (dry) changes in hydrogels (10 HEA / 0.1 BIS, 15 HEA/ 0.5 BIS, and 20 HEA / 2.0 BIS) over 100 days when incubated in LB media (37 °C, 200 rpm) with *E. coli*. **(b)** Changes in compressive moduli of hydrogels (10 HEA / 0.1 BIS, 15 HEA/ 0.5 BIS, and 20 HEA / 2.0 BIS) over 100 days when incubated in LB media (37 °C, 200 rpm) with *E. coli*. All data are presented as mean ± standard deviation (n = 3). Statistical analysis was performed by a two-tailed Student's t-test. Not significant (ns) for $P > 0.05$.

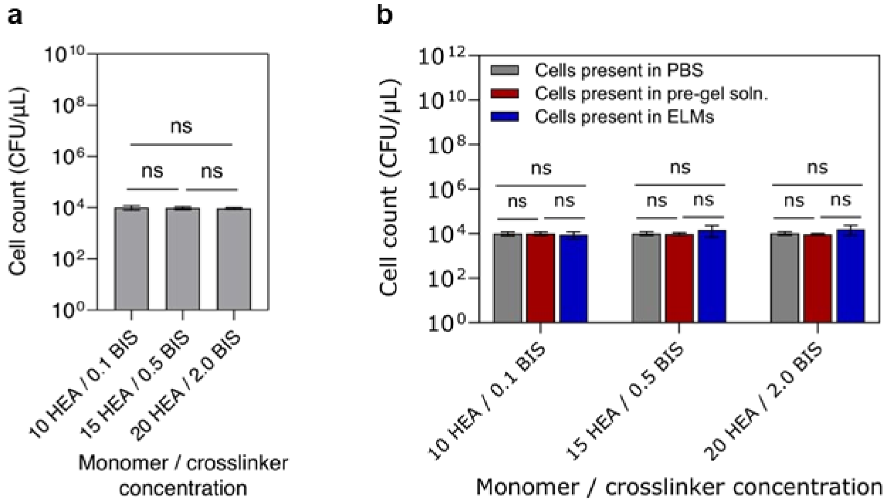

Figure S7. Cell viability during preparation of ELMs with different monomer and crosslinker concentrations (10 HEA / 0.1 BIS, 15 HEA/ 0.5 BIS, and 20 HEA / 2.0 BIS). **(a)** *E. coli* viability as a function of exposure to pregel solutions of all formulations. **(b)** Influence of the photocrosslinking process in the *E. coli* viability for all formulations. All ELMs were prepared with a cell loading of 1×10^4 cells per µL of ELM. All data are presented as mean ± standard deviation (n = 3). Statistical analysis was performed by a one-way ANOVA with post-hoc Tukey's test, not significant (ns) for $P > 0.05$.

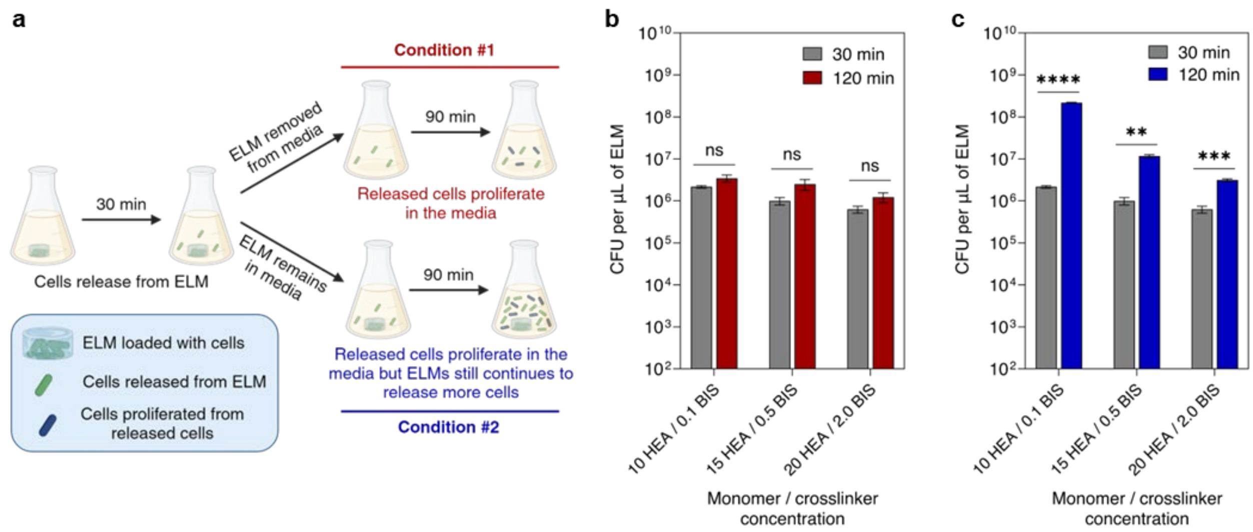

Figure S8. Influence of cell proliferation from released cells. **(a)** Illustration of cell release and further proliferation of released cells (created with BioRender.com). Cells are released from ELMs in the first 30 min. In condition #1, ELMs are removed after 30 min: the released cells continue to proliferate during the next 90 min. In condition #2, ELMs remain in the media for 2 h: both released cells continue to proliferate, and ELMs continue to release cells for the next 90 min. **(b)** Cell count in condition #1. **(c)** Cell count in condition #2. All data are presented as mean ± standard deviation (n = 3). Statistical analysis was performed by a two-tailed Student's t-test. Not significant (ns) for $P > 0.05$.

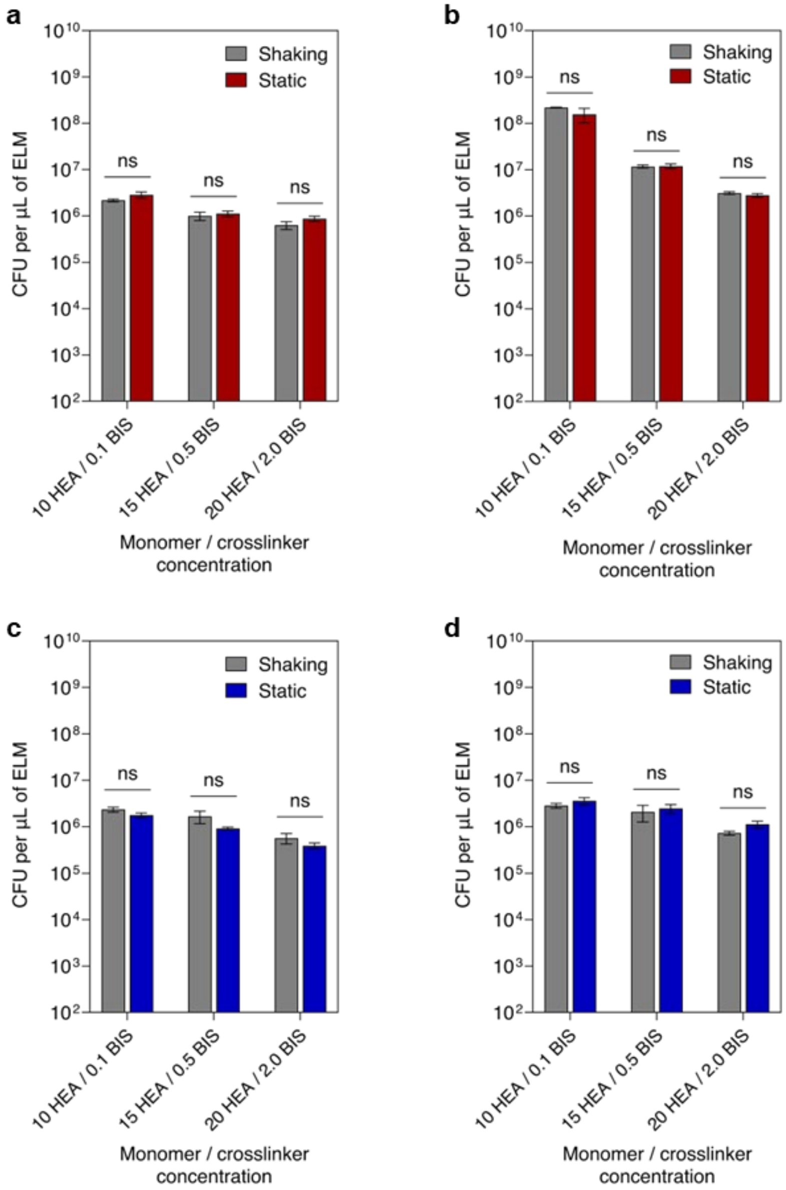

Figure S9. Comparison of cell release from ELMs in shaking (37 °C, 200 rpm) vs static conditions (37 °C). **(a)** Cell release in LB for 30 min. **(b)** Cell release in LB for 2 h. **(c)** Cell release in PBS for 30 min. **(d)** Cell release in PBS for 2 h. ELMs were prepared with a cell loading of 1 × 10^4 cells per µL of ELM and different hydrogel formulations (10 HEA / 0.1 BIS, 15 HEA / 0.5 BIS, 20 HEA / 2.0 BIS). All data are presented as mean ± standard deviation (n = 3). Statistical analysis was performed by a two-tailed Student's t-test. Not significant (ns) for $P > 0.05$.

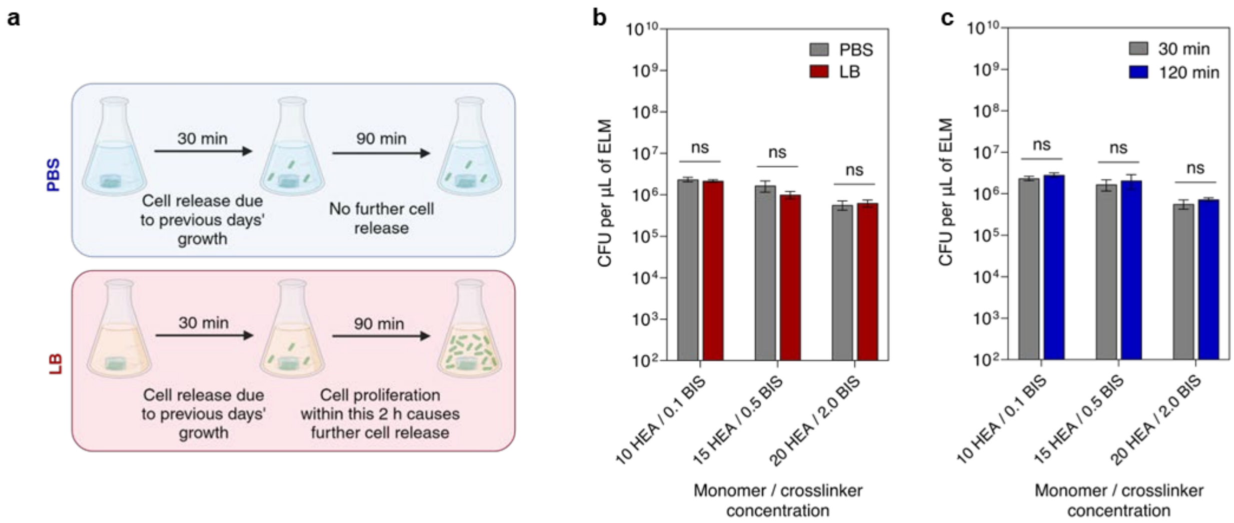

Figure S10. Comparison of cell release from ELMs in PBS and LB. (a) Schematic showing the difference in cell release from ELMs when incubated in LB vs PBS (created with BioRender.com). **(b)** Comparison of cell release from ELMs in LB and PBS for 30 min. **(c)** Comparison of cell release in PBS for 30 min and 2 h. ELMs were prepared with a cell loading of 1 × 10^4 cells per µL of ELM and different hydrogel formulations (10 HEA / 0.1 BIS, 15 HEA / 0.5 BIS, 20 HEA / 2.0 BIS). All data are presented as mean ± standard deviation (n = 3). Statistical analysis was performed by a two-tailed Student's t-test. Not significant (ns) for $P > 0.05$.

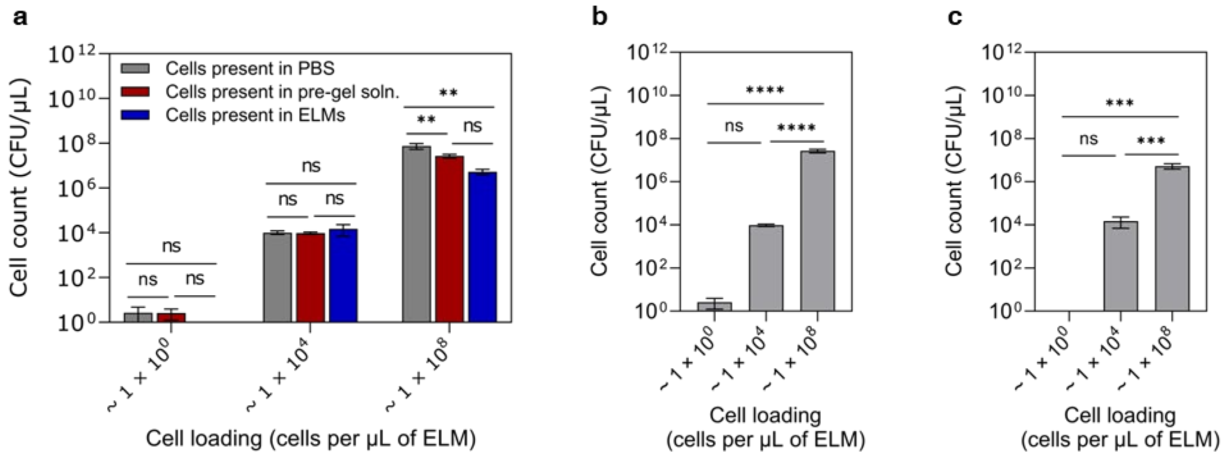

Figure S11. Cell viability during preparation of ELMs with different cell loadings (1×10^0 cells per μL of ELM, 1×10^4 cells per μL of ELM, and 1×10^8 cells per μL of ELM). **(a)** Influence of the photocrosslinking process in the *E. coli* viability when prepared with different cell loadings. **(b)** *E. coli* viability as a function of different cell loading when exposed to pregel solutions. **(c)** *E. coli* viability as a function of different cell loading after photopolymerization. All ELMs were prepared with 15 HEA / 0.5 BIS formulation. All data are presented as mean ± standard deviation ($n = 3$). Statistical analysis was performed by a one-way ANOVA with post-hoc Tukey's test, ** $P < 0.01$, *** $P < 0.001$, **** $P < 0.0001$, and not significant (ns) for $P > 0.05$.

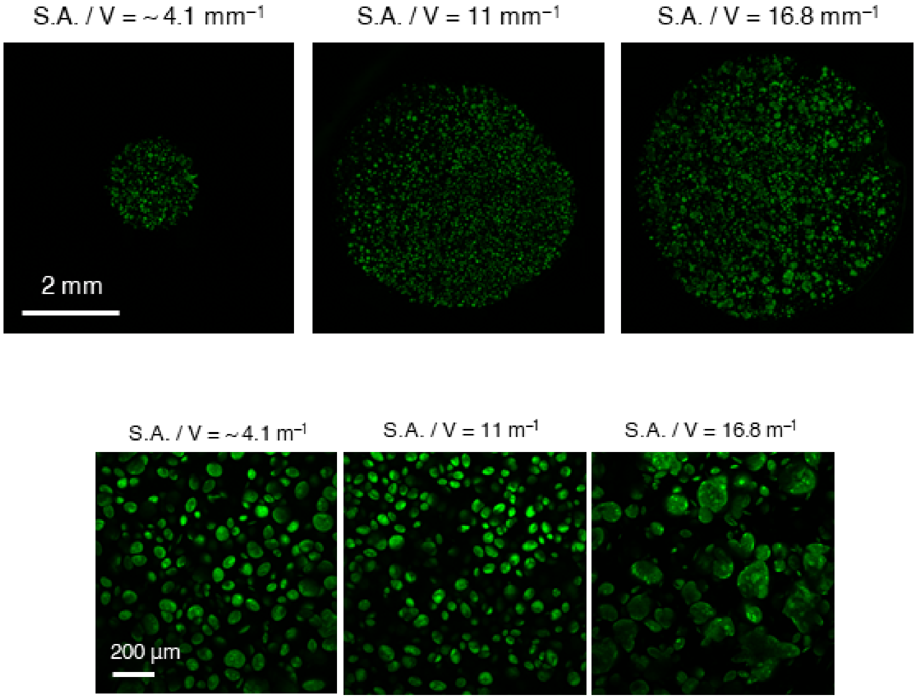

Figure S12. Microscopy images showing the differences in colony morphologies as the S.A./ V ratios are varied (~4.1, 11, and 16.8 mm^{-1}). All ELMs were prepared with medium stiffness hydrogel (15 HEA / 0.5 BIS) and a cell loading of 1 × 10^4 cells per µL of ELM.

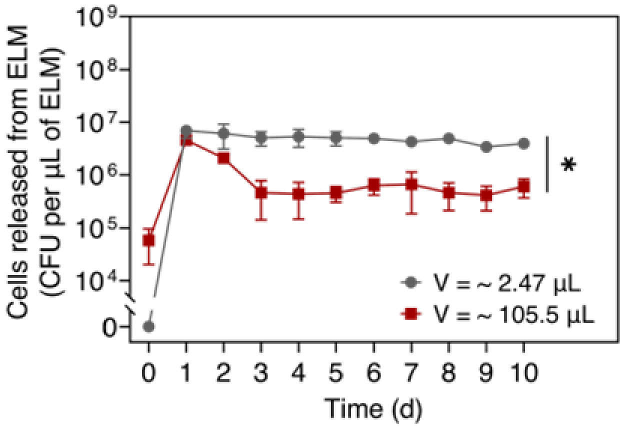

Figure S13. Cell release as a function of time from ELMs of different sizes (V = ~2.47 and ~105.5 μL). All ELMs were prepared with medium stiffness hydrogel (15 HEA / 0.5 BIS) with a cell loading of 1 × 10^4 cells per μL of ELM and had a S.A./V ratio of ~4.1 mm^{-1}. All data are presented as mean ± standard deviation (*n* = 3). Statistical analysis was performed by a two-tailed Student's *t*-test, * $P < 0.05$.

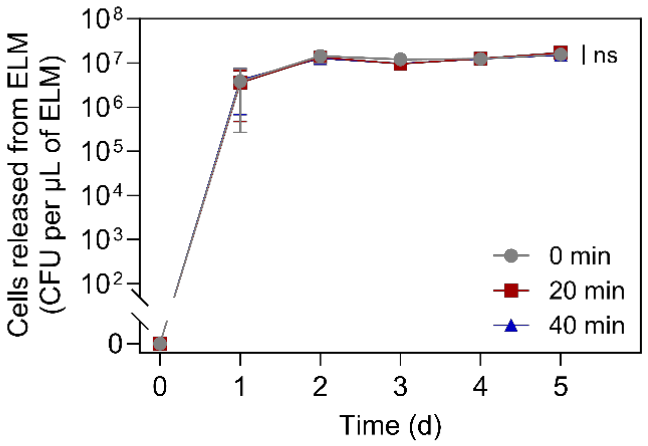

Figure S14. Sustained release of *L. paracasei* from ELMs exposed to simulated gastric fluid for 0 min, 20 min, and 40 min. All ELMs were prepared with medium stiffness hydrogel (15 HEA / 0.5 BIS) with a cell loading of 1 × 10^6 *L. paracasei* cells per µL of ELM. All data are presented as mean ± standard deviation (n = 3). Statistical analysis was performed by a one-way ANOVA with post-hoc Tukey's test, not significant (ns) for P > 0.05.
